# Supplementary figures and images for: Using viral diversity to identify HIV-1 variants under HLA-dependent selection in a systematic viral genome-wide screen
Source: PLoS Pathog. 2024 Aug 8;20(8):e1012385. doi: 10.1371/journal.ppat.1012385 (PMC11335148; doi:10.1371/journal.ppat.1012385)

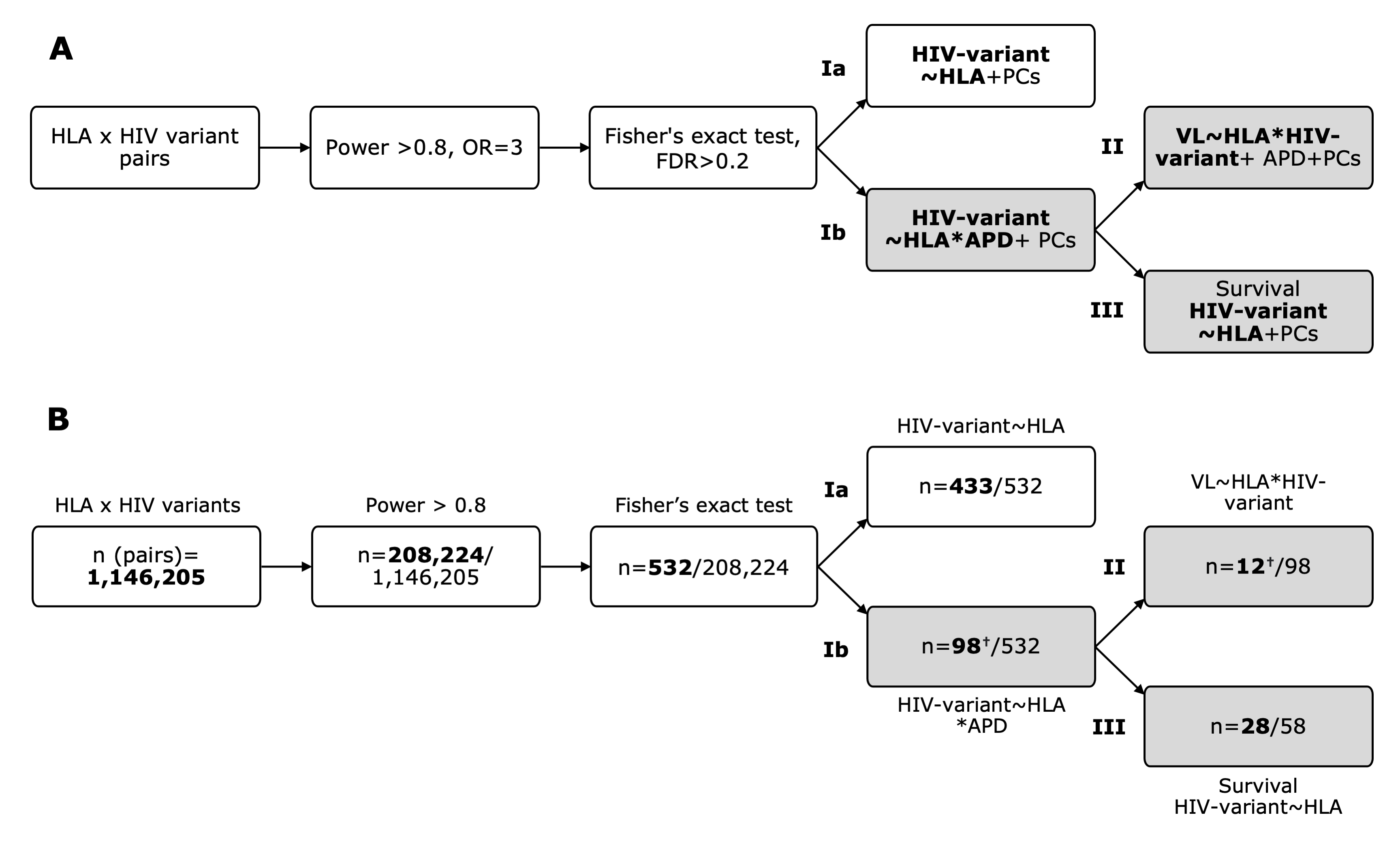

Supplement: S1 Fig — A) HLA/HIV-variant pairs were selected if power>0.8 and Fisher’s exact test p-value<0.2 (FDR-corrected). Statistical analyses performed: [Ιa] Presence of HIV variants as function of HLA alleles. [Ιb] Presence of HIV variants as function of HLA alleles, APD, and interaction between HLA and APD. [ΙΙ] VL levels as function of HLA/HIV-variant pairs (identified as significantly associated in [Ιb]); and [ΙΙΙ] longitudinal survival analysis of HLA/HIV-variant pairs identified in [Ιb]. Only samples from ART-naïve participants were used for the grey-shaded analyses ([Ιb], [ΙΙ] and [ΙΙΙ]). B) Same structure as panel A, but with the number of pairs indicated that show significance in the statistical test or have significant associations in the analyses. The format is x/y, where x stands for the number of pairs with significant associations (or † interaction terms) and y stands for the number of pairs that were included in that particular analysis. For analysis ΙΙΙ, 40 pairs were excluded from the analysis due to low sample size and lack of events, explaining the discrepancies between the y number in ΙΙ (98) and ΙΙΙ (58). (TIFF) [file ppat.1012385.s001.tiff]

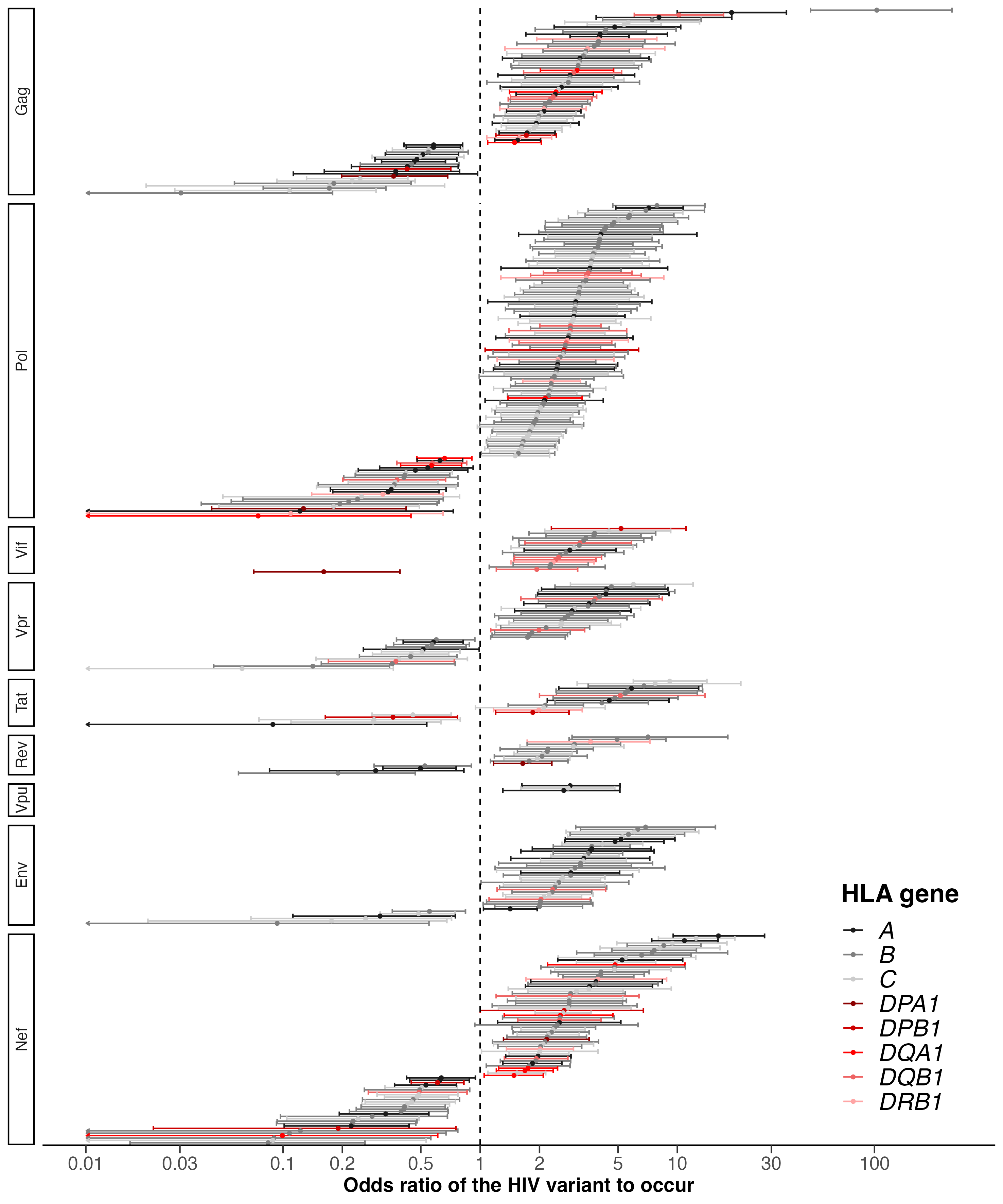

Supplement: S2 Fig — 433 pairs grouped by HIV genes and arranged by odds ratios (ORs). ORs and 95% confidence intervals derived from Fisher’s exact test. Color coding is based on HLA genes, with class Ι represented in gray and class ΙΙ represented in red. (TIFF) [file ppat.1012385.s002.tiff]

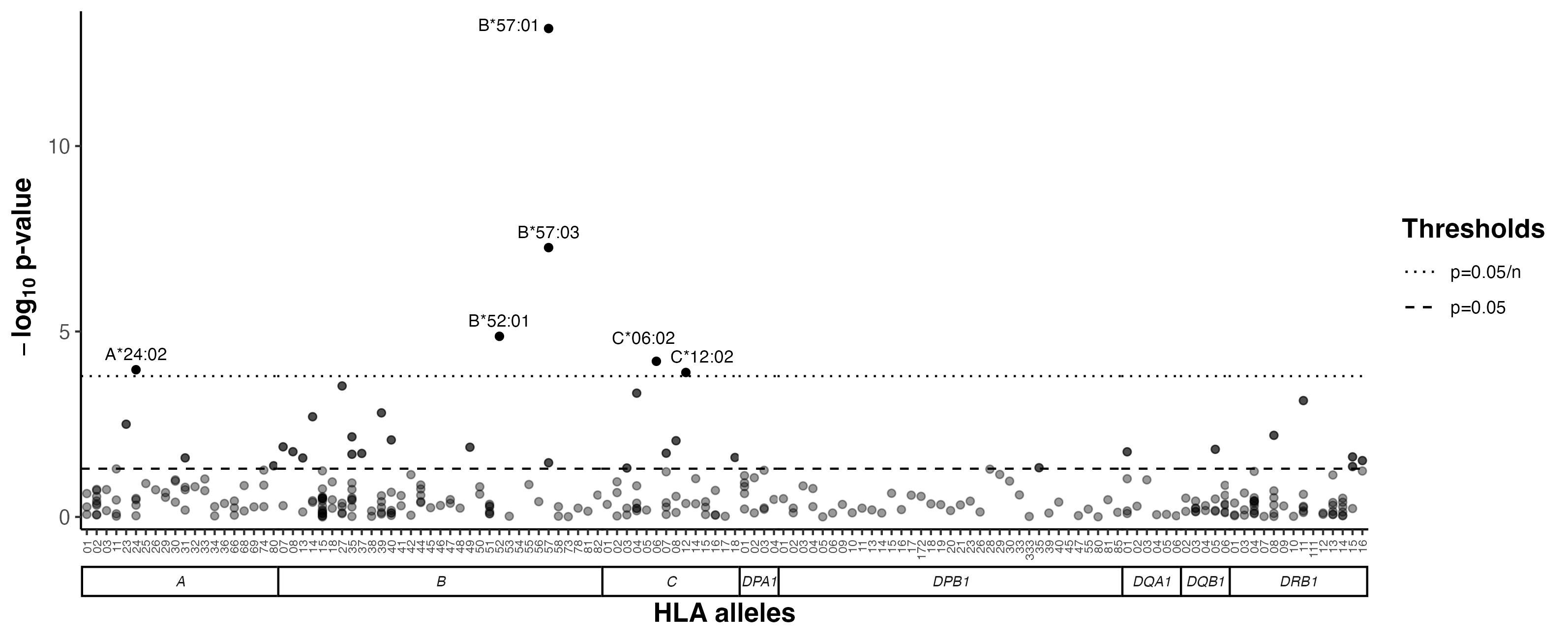

Supplement: S3 Fig — Associations between human leukocyte antigen (HLA) alleles and viral load are calculated in multivariate linear regressions and are grouped by HLA genes. P-values are shown as -log10 transformation, sample size (n) = 3,676. Two thresholds are depicted: Bonferroni threshold (p = 0.05/n; dotted line) and p = 0.05 (dashed line). (TIFF) [file ppat.1012385.s003.tiff]

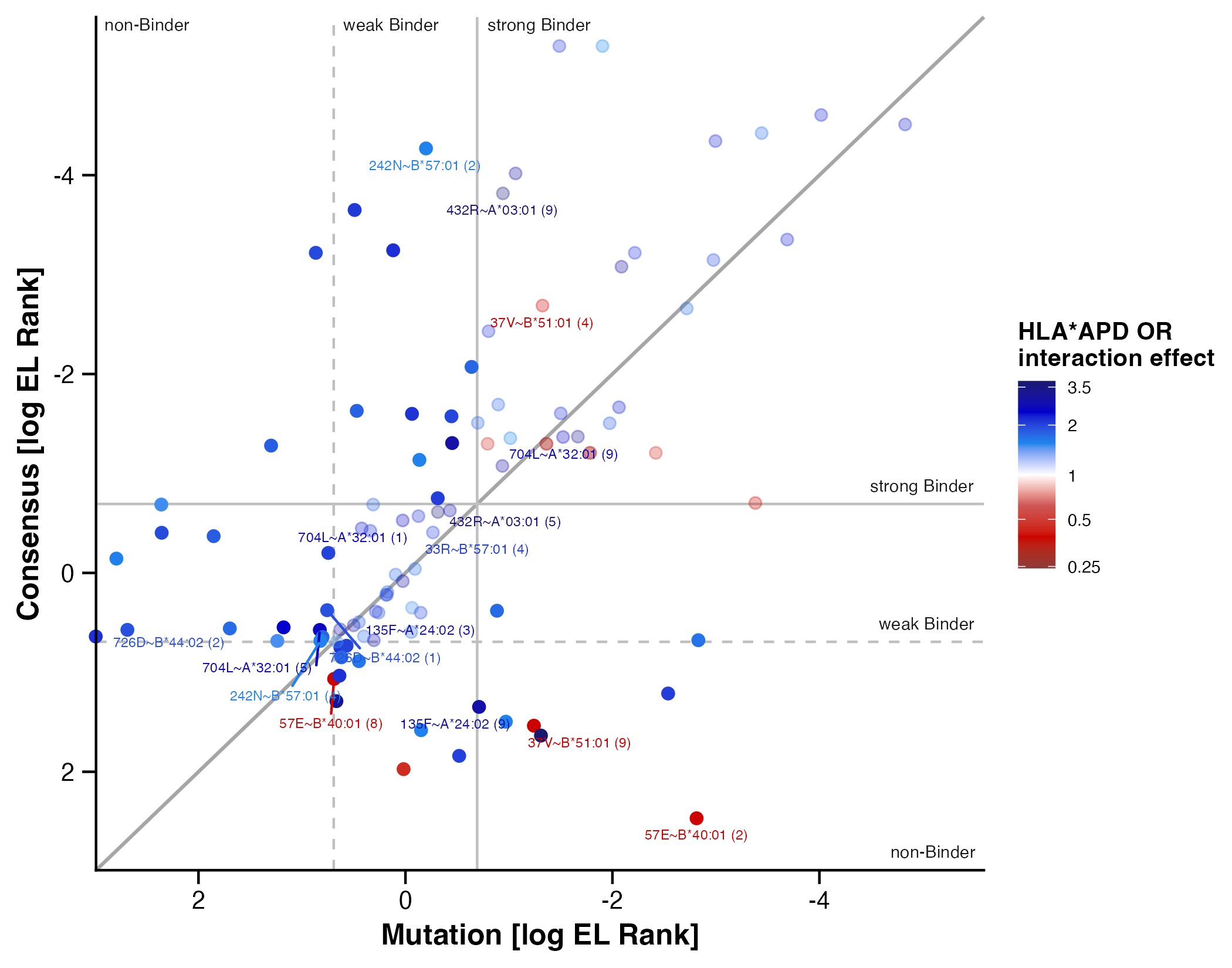

Supplement: S4 Fig — The coloration of odds ratios (OR) is derived from the HLA-APD interaction model (analysis Ιb). Pairs with no change in binding rank (strong, weak, no binding) by NetMHCpan-4.1, have lighter shading. (TIFF) [file ppat.1012385.s004.tiff]

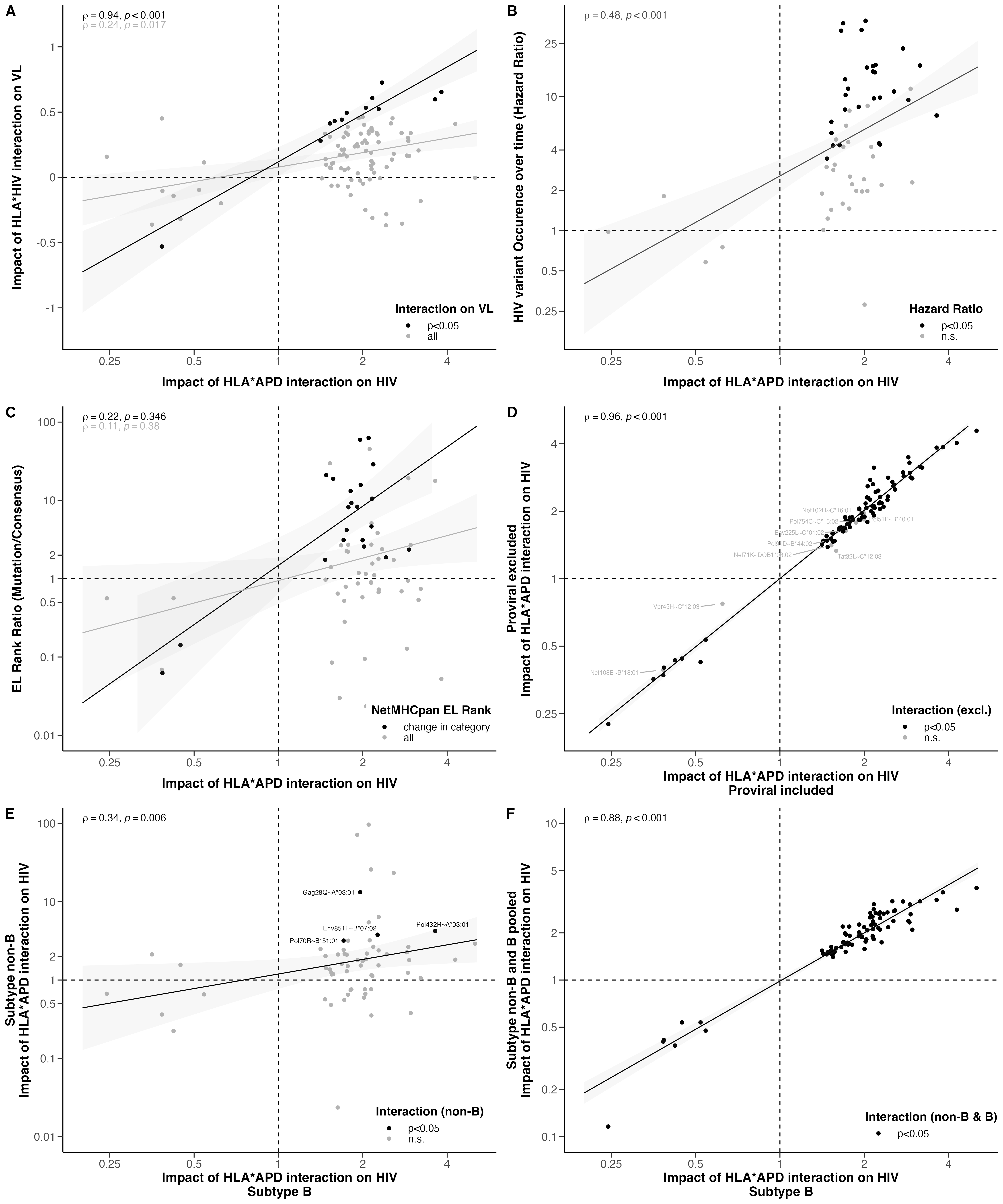

Supplement: S5 Fig — A) interaction effects of HLA/HIV-variant pairs on VL (analysis ΙΙ estimates), B) Hazard ratios of occurrence of viral variant in presence/absence of HLA (analysis ΙΙΙ cox-proportions test estimates), C) EL rank ratios (EL rank mutation/ EL rank consensus; binding predictions derived from NetMHCpan), D) Proviral sequences (n = 61) excluded, estimates of the interaction effects between HLA and APD on HIV, E) Non-B subtypes (A, C, AE, and others) estimates of the interaction effects between HLA and APD on HIV. F) All subtypes (A, B, C, AE, and others) estimates of the interaction effects between HLA and APD on HIV. Dark versus light shading indicates significance/ change on y-axis variables. Spearman’s rank correlation ρ, p-value, and linear regression lines with 95% confidence intervals were calculated either for each subgroup alone (panel A, C) or for all together (panel B, D, E, F). (TIFF) [file ppat.1012385.s005.tiff]

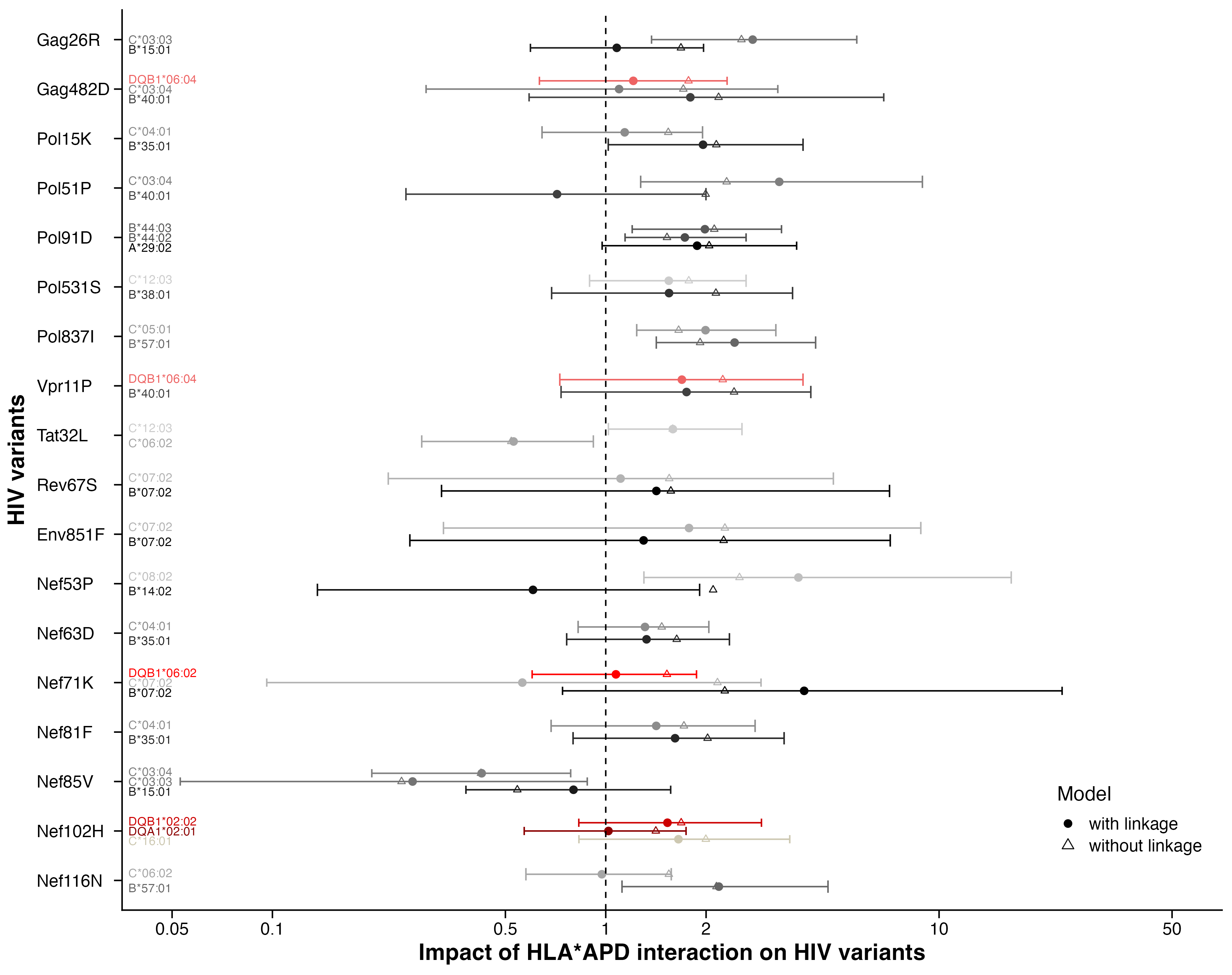

Supplement: S6 Fig — Two multivariable logistic regression models were compared—one from analysis Ιb (triangle form) and the other that includes additional interactions with other HLA alleles with the same viral variant (round form). Error bars reflect confidence intervals for the second model with linkage. Color coding is based on HLA genes, with class Ι represented in gray and class ΙΙ represented in red. (TIFF) [file ppat.1012385.s006.tiff]
