# Supplementary material for: Using viral diversity to identify HIV-1 variants under HLA-dependent selection in a systematic viral genome-wide screen
Source: PLoS Pathog. 2024 Aug 8;20(8):e1012385. doi: 10.1371/journal.ppat.1012385 (PMC11335148; doi:10.1371/journal.ppat.1012385)
Supplement: S1 Table — The sequence coverage shows the number of participants/sequences available per gene, whereas all other columns refer to the different filtering steps. See Methods for more details on procedure. (DOCX) [file ppat.1012385.s007.docx]

S1 Table: An overview of sequence coverage and number of combinations HLA and HIV variants per gene. The sequence coverage shows the number of participants/sequences available per gene, whereas all other columns refer to the different filtering steps. See Methods for more details on procedure.

| **Gene** | **Sequence coverage (# of seq.)** | **Total pairs** | **Filter**  **maf, few missing*** | **Filter power>0.8** | **Filter Fisher^†^, FDR<0.2** | **HIV and HLA associations (Ia)** | **Interaction HLA-APD**  **on HIV (Ib)** | **Interaction HLA-HIV on VL (II)** |
| --- | --- | --- | --- | --- | --- | --- | --- | --- |
| *gag* | 1,462 | 233,930 | 134,196 | 22,187 | 92 | 77 | 13 | 1 |
| *pol* | 1,471 | 365,496 | 212,055 | 34,681 | 148 | 130 | 32 | 5 |
| *vif* | 1,387 | 117,122 | 71,896 | 13,709 | 23 | 20 | 5 | 1 |
| *vpr* | 1,379 | 58,404 | 36,366 | 6,427 | 44 | 37 | 6 | 0 |
| *tat* | 1,435 | 88,234 | 43,888 | 9,032 | 25 | 19 | 2 | 1 |
| *rev* | 1,429 | 97,026 | 58,025 | 10,456 | 23 | 16 | 4 | 1 |
| *vpu* | 1,425 | 65,940 | 35,280 | 7,296 | 3 | 3 | 0 | 0 |
| *env* | 1,433 | 930,696 | 463,989 | 87,947 | 47 | 41 | 10 | 1 |
| *nef* | 1,426 | 165,478 | 90,510 | 16,489 | 127 | 89 | 26 | 2 |
| Total | 1,528 | 2,122,326 | 1,146,205 | 208,224 | 532 | 433 | 98 | 12 |

*Maf: Minor allele frequency over 0.5%, less than 20% missing; ^†^Fisher’s exact test.
