# Supplementary material for: Using viral diversity to identify HIV-1 variants under HLA-dependent selection in a systematic viral genome-wide screen
Source: PLoS Pathog. 2024 Aug 8;20(8):e1012385. doi: 10.1371/journal.ppat.1012385 (PMC11335148; doi:10.1371/journal.ppat.1012385)
Supplement: S2 Table — Odds ratios (OR) for the interaction effect of HLA and APD on the viral variant, estimates (β) of the interaction effect between HLA allele and HIV variant on viral load (VL), Hazard Ratios (HR) of the occurrence of the viral variant in presence/absence of HLA, NetMHCpan EL rank binding predictions before and upon mutation in different positions (pos), and its respective HIV 9-mer consensus epitope. 35 HLA-HIV associations pairs were newly described, all others are listed in the Publication reference list and Los Alamos (LA). All ORs and estimates having p-values<0.05 or changing binding categories are marked in bold. (DOCX) [file ppat.1012385.s008.docx]

S2 Table: All 98 pairs with significant interaction effects between HLA and APD on the HIV variant. Odds ratios (OR) for the interaction effect of HLA and APD on the viral variant, estimates (β) of the interaction effect between HLA allele and HIV variant on viral load (VL), Hazard Ratios (HR) of the occurrence of the viral variant in presence/absence of HLA, NetMHCpan EL rank binding predictions before and upon mutation in different positions (pos), and its respective HIV 9-mer consensus epitope. 35 HLA-HIV associations pairs were newly described, all others are listed in the Publication reference list and Los Alamos (LA). All ORs and estimates having *p*-values<0.05 or changing binding categories are marked in bold.

| **HIV amino acid variant^#^ ~ HLA allele** | **Interaction HLA-APD**  **OR [95% CI] on HIV variant** | **Interaction HLA-HIV β [95% CI] on VL** | **Variant hazards (+/- HLA)**  **HR [95% CI]** | **EL Rank* (epitopes):**  **Consensus → Mutation** | **HIV 9-mer for EL Rank**** | **Published HLA associations, DRM**^¶^**, LA**^$^ |
| --- | --- | --- | --- | --- | --- | --- |
| Gag-K18R~A*30:01 | **3.22 [1.28, 9.51]** | -0.18 [-0.82, 0.46] | 0.00 [0.00, Inf] ^§^ | pos 1: 0.254 → 0.189 | **K**IRLRPGGK | [1], LA |
| Gag-K26R~B*15:01 | **1.68 [1.02, 2.85]** | 0.21 [-0.25, 0.67] | **35.41 [3.88, 323.56]** |  |  | [1-4]^‡,^ LA |
| Gag-K26R~C*03:03 | **2.55 [1.43, 4.84]** | 0.45 [-0.07, 0.97] | **10.93 [1.82, 65.77]** |  |  |  |
| Gag-K28Q~A*03:01 | **1.96 [1.38, 2.82]** | -0.02 [-0.32, 0.27] | **31.58 [3.92, 254.47]** | **pos 9: 0.04 → 2.378** | RLRPGGKK**K** | [2-5]^‡^, LA |
| Gag-E93D~C*03:04 | **1.47 [1.02, 2.17]** | 0.33 [-0.00, 0.67] | 2.98 [0.79, 11.22] | **pos 1: 1.982 → 3.45** | **E**VKDTKEAL |  |
| Gag-Q127P~A*11:01 | **3.16 [1.65, 6.67]** | 0.21 [-0.37, 0.78] | **17.10 [1.55, 188.63]** | **pos 4: 3.632 → 1.949** | NSS**Q**VSQNY | [4, 6]^‡^, LA |
| Gag-R264K~B*27:05 | **2.16 [1.11, 4.24]** | **0.61 [0.03, 1.19]** |  | **pos 2: 1.895 → 19.943 pos 3: 2.812 → 1.893** | K**R**WIILGLN YK**R**WIILGL | [2-4, 7]^†^, LA |
| Gag-E398D~C*02:02 | **2.76 [1.26, 6.39]** | -0.35 [-1.08, 0.37] |  |  |  |  |
| Gag-T427N~C*02:02 | **2.54 [1.07, 6.62]** | 0.14 [-0.75, 1.02] |  |  |  |  |
| Gag-I479R~B*07:02 | **1.81 [1.16, 2.86]** | -0.14 [-0.57, 0.29] | 2.22 [0.22, 22.48] | **pos 9: 0.278 → 3.664** | TPSQKQEP**I** |  |
| Gag-E482D~B*40:01 | **2.18 [1.36, 3.59]** | 0.11 [-0.31, 0.52] | **15.21 [2.78, 83.20]** | **pos 2: 0.039 → 1.126** pos 8: 0.655 → 1.403 | K**E**LYPLASL KQEPIDK**E**L | [4, 8]^‡^, LA |
| Gag-E482D~C*03:04 | **1.71 [1.10, 2.66]** | 0.18 [-0.22, 0.57] | **13.50 [2.46, 73.99]** | **pos 8: 1.748 → 5.459** | KQEPIDK**E**L |  |
| Gag-E482D~DQB1*06:04 | **1.77 [1.04, 3.06]** | -0.07 [-0.56, 0.42] | 7.88 [0.91, 67.91] |  |  |  |
| Pol-P9L~A*31:01 | **2.34 [1.12, 4.87]** | **0.73 [0.11, 1.34]** | 0.00 [0.00, Inf] ^§^ | pos 4: 0.639 → 1.526 | LAF**P**QGKAR |  |
| Pol-E15K~B*35:01 | **2.15 [1.28, 3.72]** | 0.13 [-0.31, 0.56] | **15.43 [1.60, 148.31]** | pos 8: 0.04 → 0.109 | FPQGKAR**E**F | [9], LA |
| Pol-E15K~C*04:01 | **1.54 [1.02, 2.33]** | 0.07 [-0.30, 0.44] | 5779235924.14 [0.00, Inf] ^§^ | pos 8: 0.258 → 0.363 | FPQGKAR**E**F | [10], LA |
| Pol-T51A~DQB1*03:03 | **0.35 [0.13, 0.80]** | -0.36 [-0.91, 0.19] | 0.00 [0.00, Inf] ^§^ |  |  |  |
| Pol-T51P~B*40:01 | **1.99 [1.08, 3.76]** | 0.27 [-0.23, 0.76] | 2.42 [0.25, 23.28] | pos 6: 1.246 → 1.196 pos 4: 1.492 → 0.863 | ADRQG**T**VSF RQG**T**VSFSF | [11, 12], LA |
| Pol-T51P~C*03:04 | **2.31 [1.33, 4.10]** | -0.01 [-0.48, 0.47] | 2.19 [0.23, 21.16] |  |  |  |
| Pol-T51S~B*40:01 | **1.92 [1.05, 3.51]** | 0.24 [-0.25, 0.72] |  | pos 6: 1.246 → 1.206 pos 4: 1.492 → 1.297 | ADRQG**T**VSF RQG**T**VSFSF | [11, 12], LA |
| Pol-F54L~C*12:03 | **1.75 [1.18, 2.63]** | **0.49 [0.11, 0.88]** | 4.58 [0.50, 41.93] | **pos 1: 0.126 → 0.528 pos 4: 1.906 → 2.229 pos 2: 2.337 → 1.857** | **F**SFPQITLW TVS**F**SFPQI S**F**SFPQITL | [13], LA |
| Pol-L66I~B*13:02 | **2.07 [1.06, 4.09]** | 0.26 [-0.32, 0.85] |  |  |  | DRM |
| Pol-T68P~B*51:01 | **3.81 [1.87, 8.59]** | **0.65 [0.08, 1.23]** |  | **pos 2: 5.134 → 0.27** | V**T**IKIGGQL | [4, 14, 15]^†^, LA |
| Pol-K70R~B*51:01 | **1.71 [1.08, 2.71]** | 0.31 [-0.12, 0.74] | **8.02 [1.34, 48.10]** |  |  | [4]^‡^ |
| Pol-E91D~A*29:02 | **2.04 [1.12, 4.05]** | -0.25 [-0.82, 0.32] | **16.52 [1.84, 148.36]** |  |  | [16], LA |
| Pol-E91D~B*44:02 | **1.53 [1.02, 2.30]** | **0.41 [0.05, 0.77]** | **5.35 [1.02, 28.04]** | pos 2: 0.005 → 0.149 | E**E**MNLPGRW | [3, 4, 16]^‡^, LA |
| Pol-E91D~B*44:03 | **2.12 [1.33, 3.51]** | -0.02 [-0.43, 0.40] | 4.22 [0.85, 21.08] | pos 2: 0.005 → 0.226 | E**E**MNLPGRW | [4, 16]^‡^, LA |
| Pol-V133I~B*15:01 | **1.52 [1.05, 2.22]** | 0.07 [-0.29, 0.44] | 1.43 [0.17, 11.89] |  |  | DRM |
| Pol-I290T~B*51:01 | **1.97 [1.32, 3.02]** | 0.34 [-0.00, 0.67] | 1.96 [0.54, 7.09] | **pos 9: 0.668 → 10.547** | YTAFTIPS**I** | [2-4, 17]^‡^, LA |
| Pol-T320I~B*07:02 | **4.28 [2.14, 9.19]** | 0.41 [-0.21, 1.03] |  |  |  | [4]^‡^, LA |
| Pol-D332E~B*35:01 | **1.67 [1.05, 2.69]** | 0.07 [-0.29, 0.43] | 1.59 [0.33, 7.72] | pos 3: 0.012 → 0.032 pos 5: 0.961 → 0.912 | NP**D**IVIYQY KQNP**D**IVIY | [4, 18]^‡^, LA |
| Pol-V400E~B*57:01 | **2.10 [1.23, 3.81]** | 0.34 [-0.15, 0.82] | 0.00 [0.00, Inf] ^§^ | **pos 2: 0.026 → 1.634** | I**V**LPEKDSW | [2, 4, 19, 20]^‡^, LA |
| Pol-K432R~A*03:01 | **3.62 [2.09, 6.94]** | **0.60 [0.22, 0.98]** | **7.24 [1.85, 28.26]** | pos 9: 0.022 → 0.39 pos 5: 0.542 → 0.731 | QIYPGIKV**K** GIKV**K**QLCK | [2-4, 21]^‡^, LA |
| Pol-A531S~B*38:01 | **2.14 [1.05, 4.55]** | 0.09 [-0.51, 0.69] |  | pos 5: 1.473 → 1.332 | VQKI**A**TESI |  |
| Pol-A531S~C*12:03 | **1.77 [1.12, 2.84]** | 0.33 [-0.07, 0.72] |  | pos 2: 0.503 → 1.366 | I**A**TESIVIW | [4, 14]^‡^, LA |
| Pol-E554D~A*32:01 | **1.75 [1.03, 3.00]** | 0.16 [-0.35, 0.67] | **11.48 [1.03, 127.77]** | pos 7: 0.221 → 0.497 | IQKETW**E**AW | [4, 22]^‡^, LA |
| Pol-S623P~A*01:01 | **1.43 [1.00, 2.04]** | -0.01 [-0.31, 0.29] | 1.01 [0.12, 8.41] |  |  |  |
| Pol-E725D~B*44:03 | **2.42 [1.36, 4.47]** | -0.37 [-0.87, 0.14] | 0.00 [0.00, Inf] ^§^ | **pos 1: 1.729 → 3.246** | **E**EHEKYHSN | [4, 16]^‡^, LA |
| Pol-E726D~B*44:02 | **1.91 [1.25, 3.01]** | 0.25 [-0.10, 0.61] | **8.41 [2.08, 34.04]** | **pos 2: 1.773 → 14.689 pos 1: 1.455 → 2.131** | E**E**HEKYHSN **E**HEKYHSNW | [3, 4, 16, 23]^‡^, LA |
| Pol-S754C~C*15:02 | **1.87 [1.04, 3.48]** | 0.24 [-0.26, 0.73] | 1.98 [0.22, 17.60] |  |  |  |
| Pol-T827I~C*06:02 | **2.14 [1.20, 3.93]** | 0.19 [-0.37, 0.75] |  | **pos 7: 2.117 → 1.875** | GRWPVK**T**IH |  |
| Pol-S834P~C*05:01 | **2.75 [1.60, 4.77]** | 0.34 [-0.11, 0.78] | **22.96 [2.37, 222.02]** | pos 6: 1.968 → 1.357 | HTDNG**S**NFT | [3, 4, 24]^†^, LA |
| Pol-T837I~B*57:01 | **1.92 [1.15, 3.27]** | 0.03 [-0.45, 0.51] | 0.00 [0.00, Inf] ^§^ |  |  | [4, 16]^‡^, LA |
| Pol-T837I~C*05:01 | **1.65 [1.05, 2.63]** | 0.17 [-0.20, 0.54] | **31.13 [6.58, 147.27]** | **pos 9: 1.968 → 0.059** | HTDNGSNF**T** | [2-4, 16, 25]^‡^, LA |
| Pol-G908E~B*27:05 | **2.20 [1.23, 4.06]** | 0.04 [-0.54, 0.61] | **17.29 [2.41, 123.93]** | pos 8: 0.201 → 0.223 | KRKGGIG**G**Y | [4, 10]^‡^, LA |
| Vif-R19K~B*27:05 | **1.82 [1.03, 3.28]** | -0.02 [-0.56, 0.52] |  | **pos 2: 0.691 → 6.382** pos 6: 1.639 → 1.568 pos 4: 0.222 → 0.139 | I**R**TWKSLVK DRMRI**R**TWK MRI**R**TWKSL | [26], LA |
| Vif-K33R~B*57:01 | **2.05 [1.24, 3.69]** | **0.53 [0.11, 0.96]** | 1.98 [0.39, 10.16] | pos 4: 0.666 → 0.767 | YIS**K**KAKGW | [3], LA |
| Vif-K36R~A*03:01 | **1.78 [1.26, 2.53]** | 0.07 [-0.23, 0.36] | 2.53 [0.57, 11.34] | **pos 9: 0.196 → 1.601** | HMYISKKA**K** | [27], LA |
| Vif-R41K~A*32:01 | **1.74 [1.07, 3.00]** | 0.34 [-0.07, 0.76] | 0.00 [0.00, Inf] ^§^ | pos 6: 1.017 → 1.1 pos 8: 1.892 → 1.495 | KGWVY**R**HHY KAKGWVY**R**H |  |
| Vif-Q178R~B*14:02 | **2.16 [1.18, 4.24]** | -0.31 [-0.86, 0.24] |  | pos 7: 0.565 → 0.882 | DRWNKP**Q**KT |  |
| Vpr-Q11P~B*40:01 | **2.43 [1.16, 5.17]** | -0.28 [-0.93, 0.37] |  |  |  | [13], LA |
| Vpr-Q11P~DQB1*06:04 | **2.24 [1.09, 4.71]** | 0.34 [-0.32, 1.00] |  |  |  |  |
| Vpr-P37V~B*51:01 | **0.39 [0.14, 0.83]** | -0.10 [-0.58, 0.38] | 1.81 [0.20, 16.24] | pos 4: 0.068 → 0.266 **pos 9: 4.649 → 0.289** | FPR**P**WLHGL EAVRHFPR**P** | [28, 29], LA |
| Vpr-Y45H~C*12:03 | **0.62 [0.38, 0.97]** | -0.20 [-0.57, 0.17] | 0.75 [0.09, 5.92] |  |  |  |
| Vpr-F63M~B*13:02 | **2.06 [1.04, 4.18]** | 0.20 [-0.30, 0.71] | 8.56 [0.78, 94.52] | **pos 5: 0.472 → 0.732 pos 2: 3.363 → 0.079** | AIIR**F**LQQL R**F**LQQLLFI |  |
| Vpr-F63T~B*15:01 | **1.63 [1.02, 2.61]** | -0.06 [-0.50, 0.37] | 0.00 [0.00, Inf] ^§^ | **pos 5: 2.429 → 1.568** | AIIR**F**LQQL |  |
| Tat-F32L~C*06:02 | **0.52 [0.27, 0.89]** | -0.10 [-0.48, 0.29] | 0.00 [0.00, Inf] ^§^ |  |  |  |
| Tat-F32L~C*12:03 | **1.59 [1.03, 2.52]** | **0.43 [0.06, 0.80]** | 4.79 [0.53, 42.84] |  |  | [3, 30], LA |
| Rev-T15A~B*57:01 | **1.77 [1.10, 2.94]** | 0.39 [-0.04, 0.81] | 1.46 [0.25, 8.54] | pos 2: 0.184 → 0.408 pos 1: 1.211 → 1.191 | K**T**VRLIKLL **T**VRLIKLLY | [31], LA |
| Rev-G57E~B*40:01 | **0.38 [0.15, 0.79]** | **-0.53 [-1.00, -0.06]** | 0.00 [0.00, Inf] ^§^ | **pos 8: 2.903 → 1.992 pos 2: 11.797 → 0.06** | RQIRSIS**G**W S**G**WILSTYL |  |
| Rev-P67S~B*07:02 | **1.57 [1.09, 2.27]** | 0.11 [-0.22, 0.44] | 3.11 [0.52, 18.66] | **pos 2: 0.866 → 16.333 pos 1: 4.864 → 0.86** | R**P**AEPVPLQ **P**AEPVPLQL | [32], LA |
| Rev-P67S~C*07:02 | **1.55 [1.08, 2.23]** | 0.10 [-0.22, 0.43] | 2.84 [0.47, 17.02] | **pos 3: 0.321 → 0.874 pos 1: 4.466 → 0.379** | GR**P**AEPVPL **P**AEPVPLQL |  |
| Env-H105Q~B*38:01 | **2.91 [1.37, 7.53]** | 0.28 [-0.28, 0.84] | 0.00 [0.00, Inf] ^§^ | pos 2: 0.018 → 0.345 pos 3: 0.534 → 0.651 | M**H**EDIISLW QM**H**EDIISL | [16], LA |
| Env-I225L~C*01:02 | **1.72 [1.05, 2.85]** | 0.06 [-0.42, 0.53] | 6.07 [0.54, 68.36] | pos 8: 0.07 → 0.066 **pos 9: 1.463 → 0.413** | CAPAGFA**I**L YCAPAGFA**I** | [29], LA |
| Env-K421R~B*57:01 | **1.94 [1.16, 3.31]** | 0.45 [-0.01, 0.92] |  | pos 3: 0.043 → 0.051 | RI**K**QIINMW |  |
| Env-M426L~A*32:01 | **2.28 [1.32, 4.08]** | **0.52 [0.08, 0.97]** | 0.00 [0.00, Inf] ^§^ | pos 8: 0.011 → 0.008 | RIKQIIN**M**W | [2, 33], LA |
| Env-L565M~C*15:02 | **2.97 [1.59, 6.07]** | 0.34 [-0.07, 0.75] | 2.29 [0.28, 18.98] | pos 9: 0.046 → 0.124 pos 6: 0.341 → 0.392 pos 4: 1.765 → 1.879 | RAIEAQQH**L** EAQQH**L**LQL QQH**L**LQLTV | [34], LA |
| Env-K588Q~C*08:02 | **2.03 [1.18, 3.63]** | 0.28 [-0.22, 0.79] |  | **pos 2: 6.296 → 0.595** | L**K**DQQLLGI |  |
| Env-I704L~A*32:01 | **2.58 [1.36, 4.98]** | 0.18 [-0.37, 0.73] |  | pos 1: 0.59 → 1.027 **pos 5: 1.775 → 2.288** pos 9: 0.255 → 0.218 | **I**VNRVRQGY TVLS**I**VNRV RIVFTVLS**I** | [14], LA |
| Env-L815F~A*02:01 | **1.48 [1.09, 2.03]** | 0.10 [-0.17, 0.36] | 1.23 [0.38, 4.01] | **pos 2: 0.503 → 10.585** pos 3: 1.42 → 0.938 | L**L**NATAIAV SL**L**NATAIA | [35], LA |
| Env-L851F~B*07:02 | **2.26 [1.48, 3.51]** | 0.33 [-0.01, 0.67] | **4.50 [1.37, 14.78]** | pos 9: 0.013 → 0.05 | IPRRIRQG**L** | [2, 11, 12], LA |
| Env-L851F~C*07:02 | **2.28 [1.50, 3.52]** | 0.27 [-0.07, 0.61] | **4.40 [1.34, 14.46]** | pos 5: 0.189 → 0.127 | IRQG**L**ERAL | [11, 12], LA |
| Nef-A53P~B*14:02 | **2.10 [1.10, 4.05]** | 0.35 [-0.26, 0.96] |  |  |  | [4]^‡^ |
| Nef-A53P~C*08:02 | **2.52 [1.40, 4.69]** | 0.41 [-0.14, 0.97] |  |  |  |  |
| Nef-E63D~B*35:01 | **1.63 [1.05, 2.57]** | 0.24 [-0.10, 0.58] | **4.34 [1.29, 14.62]** |  |  | [4]^†^ |
| Nef-E63D~C*04:01 | **1.47 [1.04, 2.09]** | 0.14 [-0.14, 0.42] | **3.45 [1.08, 11.03]** | pos 4: 2 → 1.945 | AQE**E**EEVGF |  |
| Nef-R71K~B*07:02 | **2.28 [1.45, 3.69]** | 0.27 [-0.10, 0.64] | **9.84 [3.11, 31.10]** | pos 1: 0.01 → 0.018 pos 4: 0.035 → 0.025 | **R**PQVPLRPM FPV**R**PQVPL | [2-4, 32, 36]^‡^, LA |
| Nef-R71K~C*07:02 | **2.16 [1.35, 3.60]** | 0.36 [-0.04, 0.75] | **9.73 [3.08, 30.76]** |  |  | [2-4, 21, 37, 38]^†^, LA |
| Nef-R71K~DQB1*06:02 | **1.53 [1.00, 2.34]** | 0.31 [-0.05, 0.68] | **6.50 [2.05, 20.62]** |  |  | [2, 3, 39], LA |
| Nef-Y81F~B*35:01 | **2.02 [1.18, 3.58]** | 0.40 [-0.01, 0.81] | **36.96 [4.09, 333.79]** | **pos 9: 0.818 → 2.107 pos 5: 2.081 → 1.767** | QVPLRPMT**Y** RPMT**Y**KGAL | [2, 4, 10, 40]^‡^, LA |
| Nef-Y81F~C*04:01 | **1.72 [1.05, 2.88]** | 0.21 [-0.18, 0.61] | **10.30 [1.85, 57.26]** | pos 9: 1.814 → 0.946 | QVPLRPMT**Y** | [3] |
| Nef-G83A~C*03:04 | **0.45 [0.24, 0.75]** | -0.32 [-0.71, 0.06] | 0.00 [0.00, Inf] ^§^ | pos 5: 0.273 → 0.452 pos 1: 0.299 → 0.089 **pos 2: 7.195 → 1.018** | MTYK**G**ALDL **G**ALDLSHFL K**G**ALDLSHF | [3, 4, 14]^‡^, LA |
| Nef-L85F~C*08:02 | **2.90 [1.53, 5.74]** | 0.30 [-0.21, 0.82] | 0.00 [0.00, Inf] ^§^ | pos 3: 1.087 → 1.026 | GA**L**DLSHFL | [2, 4, 41]^‡^, LA |
| Nef-L85V~B*15:01 | **0.54 [0.28, 0.94]** | 0.11 [-0.43, 0.66] | 0.58 [0.13, 2.61] |  |  |  |
| Nef-L85V~C*03:03 | **0.24 [0.05, 0.72]** | 0.16 [-0.46, 0.77] | 0.98 [0.27, 3.51] | pos 7: 0.273 → 0.256 pos 3: 0.299 → 0.168 | MTYKGA**L**DL GA**L**DLSHFL | [4]^‡^ |
| Nef-L85V~C*03:04 | **0.42 [0.20, 0.77]** | -0.14 [-0.61, 0.33] | 0.00 [0.00, Inf] ^§^ | pos 7: 0.273 → 0.256 pos 3: 0.299 → 0.168 | MTYKGA**L**DL GA**L**DLSHFL | [4]^‡^ |
| Nef-K92R~A*11:01 | **2.15 [1.23, 3.91]** | 0.38 [-0.05, 0.81] | **16.95 [2.31, 124.38]** | **pos 9: 0.202 → 0.939** | ALDLSHFL**K** | [2-4, 6]^‡^, LA |
| Nef-Y102H~C*16:01 | **2.00 [1.06, 4.17]** | 0.46 [-0.08, 1.00] | 0.00 [0.00, Inf] ^§^ | **pos 1: 0.207 → 0.641** | **Y**SQKRQDIL | [4]^‡^ |
| Nef-Y102H~DQA1*02:01 | **1.41 [1.03, 1.95]** | **0.28 [0.01, 0.55]** | 1.89 [0.19, 18.29] |  |  |  |
| Nef-Y102H~DQB1*02:02 | **1.68 [1.17, 2.46]** | **0.44 [0.14, 0.75]** | 4.23 [0.44, 40.90] |  |  |  |
| Nef-D108E~B*18:01 | **0.38 [0.13, 0.85]** | 0.45 [-0.13, 1.04] | 0.00 [0.00, Inf] ^§^ | pos 2: 0.495 → 0.034 | Q**D**ILDLWVY | [4, 42]^†^, LA |
| Nef-H116N~B*57:01 | **2.15 [1.26, 4.04]** | 0.06 [-0.36, 0.49] | 3.58 [0.72, 17.90] | pos 1: 0.088 → 0.448 | **H**TQGYFPDW | [2-4, 16, 43]^‡^, LA |
| Nef-H116N~C*06:02 | **1.55 [1.11, 2.18]** | -0.06 [-0.35, 0.23] | **4.33 [1.16, 16.15]** |  |  |  |
| Nef-Q125H~B*13:02 | **5.03 [2.00, 16.25]** | -0.00 [-0.65, 0.64] |  |  |  | [4]^†^ |
| Nef-Y135F~A*24:02 | **2.87 [1.82, 4.75]** | 0.17 [-0.17, 0.51] | **9.48 [2.86, 31.40]** | pos 3: 1.695 → 1.649 **pos 9: 3.848 → 0.491** | TR**Y**PLTFGW YTPGPGTR**Y** | [2-4, 44]^‡^, LA |
| Nef-V153I~A*02:01 | **2.00 [1.31, 3.17]** | -0.05 [-0.35, 0.25] | 0.28 [0.03, 2.73] |  |  | [4]^‡^ |
| Nef-K184R~DQA1*04:01 | **2.43 [1.28, 4.86]** | 0.08 [-0.42, 0.58] | 0.00 [0.00, Inf] ^§^ |  |  |  |
| Nef-H192R~B*51:01 | **2.93 [1.79, 4.89]** | 0.16 [-0.24, 0.57] | 11.48 [0.61, 217.30] | **pos 7: 0.271 → 0.637** | DSRLAF**H**HV |  |

^#^Nomenclature of HIV amino acid variant as follows: [Gene]-[Consensus] [HXB2 gene numbering] [Mutation]; *Rank≤0.5: strong binder, rank≤2.0: weaker binder, otherwise non-binder; **mutation position in bold; ^†^indirect, and ^‡^direct effects according to [4]; ^§^were excluded from results due to low numbers per group (n=20); ^¶^drug-resistance mutation reported in [45]. LA^$^: Epitope association reported in [46].

**References for S2 Table:**

1. Pereyra F, Heckerman D, Carlson JM, Kadie C, Soghoian DZ, Karel D, et al. HIV Control Is Mediated in Part by CD8^+^T-Cell Targeting of Specific Epitopes. Journal of Virology. 2014;88(22):12937-48. doi: 10.1128/jvi.01004-14.

2. Gabrielaite M, Bennedbæk M, Zucco AG, Ekenberg C, Murray DD, Kan VL, et al. Human Immunotypes Impose Selection on Viral Genotypes Through Viral Epitope Specificity. J Infect Dis. 2021;224(12):2053-63. doi: 10.1093/infdis/jiab253. PubMed PMID: 33974707; PubMed Central PMCID: PMCPMC8672757.

3. Bartha I, Carlson JM, Brumme CJ, McLaren PJ, Brumme ZL, John M, et al. A genome-to-genome analysis of associations between human genetic variation, HIV-1 sequence diversity, and viral control. Elife. 2013;2:e01123. Epub 20131029. doi: 10.7554/eLife.01123. PubMed PMID: 24171102; PubMed Central PMCID: PMCPMC3807812.

4. Brumme ZL, John M, Carlson JM, Brumme CJ, Chan D, Brockman MA, et al. HLA-associated immune escape pathways in HIV-1 subtype B Gag, Pol and Nef proteins. PLoS One. 2009;4(8):e6687. Epub 20090819. doi: 10.1371/journal.pone.0006687. PubMed PMID: 19690614; PubMed Central PMCID: PMCPMC2723923.

5. Milicic A, Edwards CT, Hué S, Fox J, Brown H, Pillay T, et al. Sexual transmission of single human immunodeficiency virus type 1 virions encoding highly polymorphic multisite cytotoxic T-lymphocyte escape variants. J Virol. 2005;79(22):13953-62. doi: 10.1128/JVI.79.22.13953-13962.2005. PubMed PMID: 16254331; PubMed Central PMCID: PMCPMC1280182.

6. Yaciuk JC, Skaley M, Bardet W, Schafer F, Mojsilovic D, Cate S, et al. Direct interrogation of viral peptides presented by the class I HLA of HIV-infected T cells. J Virol. 2014;88(22):12992-3004. Epub 20140827. doi: 10.1128/JVI.01914-14. PubMed PMID: 25165114; PubMed Central PMCID: PMCPMC4249081.

7. Tenzer S, Crawford H, Pymm P, Gifford R, Sreenu VB, Weimershaus M, et al. HIV-1 adaptation to antigen processing results in population-level immune evasion and affects subtype diversification. Cell Rep. 2014;7(2):448-63. Epub 20140413. doi: 10.1016/j.celrep.2014.03.031. PubMed PMID: 24726370; PubMed Central PMCID: PMCPMC4005910.

8. Barton JP, Goonetilleke N, Butler TC, Walker BD, McMichael AJ, Chakraborty AK. Relative rate and location of intra-host HIV evolution to evade cellular immunity are predictable. Nat Commun. 2016;7:11660. Epub 20160523. doi: 10.1038/ncomms11660. PubMed PMID: 27212475; PubMed Central PMCID: PMCPMC4879252.

9. Friedrich D, Jalbert E, Dinges WL, Sidney J, Sette A, Huang Y, et al. Vaccine-induced HIV-specific CD8+ T cells utilize preferential HLA alleles and target-specific regions of HIV-1. J Acquir Immune Defic Syndr. 2011;58(3):248-52. doi: 10.1097/QAI.0b013e318228f992. PubMed PMID: 21709567; PubMed Central PMCID: PMCPMC3196811.

10. Almeida CA, Bronke C, Roberts SG, McKinnon E, Keane NM, Chopra A, et al. Translation of HLA-HIV associations to the cellular level: HIV adapts to inflate CD8 T cell responses against Nef and HLA-adapted variant epitopes. J Immunol. 2011;187(5):2502-13. Epub 20110805. doi: 10.4049/jimmunol.1100691. PubMed PMID: 21821798; PubMed Central PMCID: PMCPMC3183574.

11. Yang Y, Ganusov VV. Defining Kinetic Properties of HIV-Specific CD8⁺ T-Cell Responses in Acute Infection. Microorganisms. 2019;7(3). Epub 20190304. doi: 10.3390/microorganisms7030069. PubMed PMID: 30836625; PubMed Central PMCID: PMCPMC6462943.

12. Turnbull EL, Wong M, Wang S, Wei X, Jones NA, Conrod KE, et al. Kinetics of expansion of epitope-specific T cell responses during primary HIV-1 infection. J Immunol. 2009;182(11):7131-45. doi: 10.4049/jimmunol.0803658. PubMed PMID: 19454710.

13. Warren JA, Zhou S, Xu Y, Moeser MJ, MacMillan DR, Council O, et al. The HIV-1 latent reservoir is largely sensitive to circulating T cells. Elife. 2020;9. Epub 20201006. doi: 10.7554/eLife.57246. PubMed PMID: 33021198; PubMed Central PMCID: PMCPMC7593086.

14. Pereyra F, Heckerman D, Carlson JM, Kadie C, Soghoian DZ, Karel D, et al. HIV control is mediated in part by CD8+ T-cell targeting of specific epitopes. J Virol. 2014;88(22):12937-48. Epub 20140827. doi: 10.1128/JVI.01004-14. PubMed PMID: 25165115; PubMed Central PMCID: PMCPMC4249072.

15. Migueles SA, Mendoza D, Zimmerman MG, Martins KM, Toulmin SA, Kelly EP, et al. CD8(+) T-cell Cytotoxic Capacity Associated with Human Immunodeficiency Virus-1 Control Can Be Mediated through Various Epitopes and Human Leukocyte Antigen Types. EBioMedicine. 2015;2(1):46-58. Epub 20141222. doi: 10.1016/j.ebiom.2014.12.009. PubMed PMID: 26137533; PubMed Central PMCID: PMCPMC4485486.

16. Kunwar P, Hawkins N, Dinges WL, Liu Y, Gabriel EE, Swan DA, et al. Superior control of HIV-1 replication by CD8+ T cells targeting conserved epitopes: implications for HIV vaccine design. PLoS One. 2013;8(5):e64405. Epub 20130531. doi: 10.1371/journal.pone.0064405. PubMed PMID: 23741326; PubMed Central PMCID: PMCPMC3669284.

17. Ahmed T, Borthwick NJ, Gilmour J, Hayes P, Dorrell L, Hanke T. Control of HIV-1 replication in vitro by vaccine-induced human CD8(+) T cells through conserved subdominant Pol epitopes. Vaccine. 2016;34(9):1215-24. Epub 20160116. doi: 10.1016/j.vaccine.2015.12.021. PubMed PMID: 26784683; PubMed Central PMCID: PMCPMC4769096.

18. Murakoshi H, Koyanagi M, Akahoshi T, Chikata T, Kuse N, Gatanaga H, et al. Impact of a single HLA-A*24:02-associated escape mutation on the detrimental effect of HLA-B*35:01 in HIV-1 control. EBioMedicine. 2018;36:103-12. Epub 20180922. doi: 10.1016/j.ebiom.2018.09.022. PubMed PMID: 30249546; PubMed Central PMCID: PMCPMC6197679.

19. Menéndez-Arias L, Mas A, Domingo E. Cytotoxic T-lymphocyte responses to HIV-1 reverse transcriptase (review). Viral Immunol. 1998;11(4):167-81. doi: 10.1089/vim.1998.11.167. PubMed PMID: 10189185.

20. van der Burg SH, Klein MR, Pontesilli O, Holwerda AM, Drijfhout JW, Kast WM, et al. HIV-1 reverse transcriptase-specific CTL against conserved epitopes do not protect against progression to AIDS. J Immunol. 1997;159(7):3648-54. PubMed PMID: 9317165.

21. Du VY, Bansal A, Carlson J, Salazar-Gonzalez JF, Salazar MG, Ladell K, et al. HIV-1-Specific CD8 T Cells Exhibit Limited Cross-Reactivity during Acute Infection. J Immunol. 2016;196(8):3276-86. Epub 20160316. doi: 10.4049/jimmunol.1502411. PubMed PMID: 26983786; PubMed Central PMCID: PMCPMC4821763.

22. Goulder PJ, Walker BD. HIV and HLA class I: an evolving relationship. Immunity. 2012;37(3):426-40. doi: 10.1016/j.immuni.2012.09.005. PubMed PMID: 22999948; PubMed Central PMCID: PMCPMC3966573.

23. Keane NM, Roberts SG, Almeida CA, Krishnan T, Chopra A, Demaine E, et al. High-avidity, high-IFNγ-producing CD8 T-cell responses following immune selection during HIV-1 infection. Immunol Cell Biol. 2012;90(2):224-34. Epub 20110517. doi: 10.1038/icb.2011.34. PubMed PMID: 21577229; PubMed Central PMCID: PMCPMC3173576.

24. Brockman MA, Chopera DR, Olvera A, Brumme CJ, Sela J, Markle TJ, et al. Uncommon pathways of immune escape attenuate HIV-1 integrase replication capacity. J Virol. 2012;86(12):6913-23. Epub 20120411. doi: 10.1128/JVI.07133-11. PubMed PMID: 22496233; PubMed Central PMCID: PMCPMC3393549.

25. Ferrari G, Korber B, Goonetilleke N, Liu MK, Turnbull EL, Salazar-Gonzalez JF, et al. Relationship between functional profile of HIV-1 specific CD8 T cells and epitope variability with the selection of escape mutants in acute HIV-1 infection. PLoS Pathog. 2011;7(2):e1001273. Epub 20110210. doi: 10.1371/journal.ppat.1001273. PubMed PMID: 21347345; PubMed Central PMCID: PMCPMC3037354.

26. Borthwick N, Lin Z, Akahoshi T, Llano A, Silva-Arrieta S, Ahmed T, et al. Novel, in-natural-infection subdominant HIV-1 CD8+ T-cell epitopes revealed in human recipients of conserved-region T-cell vaccines. PLoS One. 2017;12(4):e0176418. Epub 20170427. doi: 10.1371/journal.pone.0176418. PubMed PMID: 28448594; PubMed Central PMCID: PMCPMC5407754.

27. Altfeld M, Kalife ET, Qi Y, Streeck H, Lichterfeld M, Johnston MN, et al. HLA Alleles Associated with Delayed Progression to AIDS Contribute Strongly to the Initial CD8(+) T Cell Response against HIV-1. PLoS Med. 2006;3(10):e403. doi: 10.1371/journal.pmed.0030403. PubMed PMID: 17076553; PubMed Central PMCID: PMCPMC1626551.

28. Liu Y, McNevin J, Cao J, Zhao H, Genowati I, Wong K, et al. Selection on the human immunodeficiency virus type 1 proteome following primary infection. J Virol. 2006;80(19):9519-29. doi: 10.1128/JVI.00575-06. PubMed PMID: 16973556; PubMed Central PMCID: PMCPMC1617227.

29. Liu Y, McNevin JP, Holte S, McElrath MJ, Mullins JI. Dynamics of viral evolution and CTL responses in HIV-1 infection. PLoS One. 2011;6(1):e15639. Epub 20110120. doi: 10.1371/journal.pone.0015639. PubMed PMID: 21283794; PubMed Central PMCID: PMCPMC3024315.

30. Cao J, McNevin J, Holte S, Fink L, Corey L, McElrath MJ. Comprehensive analysis of human immunodeficiency virus type 1 (HIV-1)-specific gamma interferon-secreting CD8+ T cells in primary HIV-1 infection. J Virol. 2003;77(12):6867-78. doi: 10.1128/jvi.77.12.6867-6878.2003. PubMed PMID: 12768006; PubMed Central PMCID: PMCPMC156203.

31. Goonetilleke N, Liu MK, Salazar-Gonzalez JF, Ferrari G, Giorgi E, Ganusov VV, et al. The first T cell response to transmitted/founder virus contributes to the control of acute viremia in HIV-1 infection. J Exp Med. 2009;206(6):1253-72. Epub 20090601. doi: 10.1084/jem.20090365. PubMed PMID: 19487423; PubMed Central PMCID: PMCPMC2715063.

32. Kløverpris HN, Adland E, Koyanagi M, Stryhn A, Harndahl M, Matthews PC, et al. HIV subtype influences HLA-B*07:02-associated HIV disease outcome. AIDS Res Hum Retroviruses. 2014;30(5):468-75. Epub 20131004. doi: 10.1089/AID.2013.0197. PubMed PMID: 24010680; PubMed Central PMCID: PMCPMC4010166.

33. Harrer T, Harrer E, Kalams SA, Barbosa P, Trocha A, Johnson RP, et al. Cytotoxic T lymphocytes in asymptomatic long-term nonprogressing HIV-1 infection. Breadth and specificity of the response and relation to in vivo viral quasispecies in a person with prolonged infection and low viral load. J Immunol. 1996;156(7):2616-23. PubMed PMID: 8786327.

34. Zhang SC, Martin E, Shimada M, Godfrey SB, Fricke J, Locastro S, et al. Aminopeptidase substrate preference affects HIV epitope presentation and predicts immune escape patterns in HIV-infected individuals. J Immunol. 2012;188(12):5924-34. Epub 20120514. doi: 10.4049/jimmunol.1200219. PubMed PMID: 22586036; PubMed Central PMCID: PMCPMC3370117.

35. Peter K, Men Y, Pantaleo G, Gander B, Corradin G. Induction of a cytotoxic T-cell response to HIV-1 proteins with short synthetic peptides and human compatible adjuvants. Vaccine. 2001;19(30):4121-9. doi: 10.1016/s0264-410x(01)00179-7. PubMed PMID: 11457536.

36. Carlson JM, Du VY, Pfeifer N, Bansal A, Tan VY, Power K, et al. Impact of pre-adapted HIV transmission. Nat Med. 2016;22(6):606-13. Epub 20160516. doi: 10.1038/nm.4100. PubMed PMID: 27183217; PubMed Central PMCID: PMCPMC4899163.

37. Carlson JM, Schaefer M, Monaco DC, Batorsky R, Claiborne DT, Prince J, et al. HIV transmission. Selection bias at the heterosexual HIV-1 transmission bottleneck. Science. 2014;345(6193):1254031. Epub 20140710. doi: 10.1126/science.1254031. PubMed PMID: 25013080; PubMed Central PMCID: PMCPMC4289910.

38. Zhang Y, Chikata T, Kuse N, Murakoshi H, Gatanaga H, Oka S, et al. Immunological Control of HIV-1 Disease Progression by Rare Protective HLA Allele. J Virol. 2022;96(22):e0124822. Epub 20221103. doi: 10.1128/jvi.01248-22. PubMed PMID: 36326273; PubMed Central PMCID: PMCPMC9683060.

39. Li W, Li C, Xia W, Li X. HLA-DQB1*06 and breadth of Nef core region-specific T-cell response are associated with slow disease progression in antiretroviral therapy-naive Chinese HIV-1 subtype B patients. Hum Vaccin Immunother. 2017;13(10):2341-7. doi: 10.1080/21645515.2017.1340138. PubMed PMID: 28771107; PubMed Central PMCID: PMCPMC5647954.

40. Tomiyama H, Oka S, Ogg GS, Ida S, McMichael AJ, Takiguchi M. Expansion of HIV-1-specific CD28- CD45RA- CD8+ T cells in chronically HIV-1-infected individuals. AIDS. 2000;14(13):2049-51. doi: 10.1097/00002030-200009080-00023. PubMed PMID: 10997412.

41. Nixon DF, Douek D, Kuebler PJ, Jin X, Vesanen M, Bonhoeffer S, et al. Molecular tracking of an Human Immunodeficiency Virus nef specific cytotoxic T-cell clone shows persistence of clone-specific T-cell receptor DNA but not mRNA following early combination antiretroviral therapy. Immunol Lett. 1999;66(1-3):219-28. doi: 10.1016/s0165-2478(98)00162-x. PubMed PMID: 10203058.

42. Karlsson I, Kløverpris H, Jensen KJ, Stryhn A, Buus S, Karlsson A, et al. Identification of conserved subdominant HIV Type 1 CD8(+) T Cell epitopes restricted within common HLA Supertypes for therapeutic HIV Type 1 vaccines. AIDS Res Hum Retroviruses. 2012;28(11):1434-43. Epub 20120814. doi: 10.1089/aid.2012.0081. PubMed PMID: 22747336.

43. Lécuroux C, Sáez-Cirión A, Girault I, Versmisse P, Boufassa F, Avettand-Fenoël V, et al. Both HLA-B*57 and plasma HIV RNA levels contribute to the HIV-specific CD8+ T cell response in HIV controllers. J Virol. 2014;88(1):176-87. Epub 20131016. doi: 10.1128/JVI.02098-13. PubMed PMID: 24131719; PubMed Central PMCID: PMCPMC3911721.

44. Han C, Kawana-Tachikawa A, Shimizu A, Zhu D, Nakamura H, Adachi E, et al. Switching and emergence of CTL epitopes in HIV-1 infection. Retrovirology. 2014;11:38. Epub 20140521. doi: 10.1186/1742-4690-11-38. PubMed PMID: 24886641; PubMed Central PMCID: PMCPMC4036671.

45. Wensing AM, Calvez V, Ceccherini-Silberstein F, Charpentier C, Günthard HF, Paredes R, et al. 2022 update of the drug resistance mutations in HIV-1. Top Antivir Med. 2022;30(4):559-74. PubMed PMID: 36375130; PubMed Central PMCID: PMCPMC9681141.

46. Los Alamos Immunology database [cited 2024 04]. Available from: <https://www.hiv.lanl.gov/content/immunology/maps/maps.html>.
