# Supplementary material for: Using viral diversity to identify HIV-1 variants under HLA-dependent selection in a systematic viral genome-wide screen
Source: PLoS Pathog. 2024 Aug 8;20(8):e1012385. doi: 10.1371/journal.ppat.1012385 (PMC11335148; doi:10.1371/journal.ppat.1012385)
Supplement: S3 Table — The number of participants used for cross-sectional (Ι and ΙΙ) and longitudinal analysis (ΙΙΙ) is shown for each analysis, with further information on the percentage of subtype B and non-B and the percentage of ART-naïve participants in the study population. (DOCX) [file ppat.1012385.s009.docx]

S3 Table: Characteristics of SHCS participants included. The number of participants used for cross-sectional (I and II) and longitudinal analysis (III) is shown for each analysis, with further information on the percentage of subtype B and non-B and the percentage of ART-naïve participants in the study population.

|  | **Analysis Ia** | **Analysis Ib** | **Analysis II** | **Analysis III** |
| --- | --- | --- | --- | --- |
| **Sample type** | HLA, HIV seq | HLA, HIV seq | VL, HLA, HIV seq | HLA, HIV seq |
| **Number of SHCS participants** | 2039 | 1364 | 1072 | 152 |
| **Subtype:** n (%) |  |  |  |  |
| B | 1528 (74.9) | **1044** (76.5) | **829** (77.3) | **130** (85.5) |
| non-B | 511 (25.1) | 320 (23.5) | 243 (22.7) | 22 (14.5) |
| **ART-naïve:** n (%) | 1364 (66.9) | 1364 (100.0) | 1072 (100.0) | 152 (100.0) |
