# Supplementary material for: Using viral diversity to identify HIV-1 variants under HLA-dependent selection in a systematic viral genome-wide screen
Source: PLoS Pathog. 2024 Aug 8;20(8):e1012385. doi: 10.1371/journal.ppat.1012385 (PMC11335148; doi:10.1371/journal.ppat.1012385)
Supplement: Attachment — Submitted filename: letter_responses_review.docx [file ppat.1012385.s011.docx]

Dear Editor

We submit herewith a revised version of our manuscript. We are grateful for the reviewers' constructive comments, which we addressed in detail in our point-by-point reply below.

Throughout the point-to-point reply, we use the following color code:

R: Our responses are marked in blue.

Line x-y: Modifications to the manuscript text are marked in red.

Thank you for reconsidering our paper for publication in PLoS Pathogens.

On behalf of all authors,

Nadia Neuner-Jehle and Roger Kouyos

Each of the reviewers focused on a different issue in your paper. You should carefully consider each of these comments in a revision.

**Part I - Summary**
Please use this section to discuss strengths/weaknesses of study, novelty/significance, general execution and scholarship.

Reviewer #1: Neuner-Jehle and colleagues conducted a large scale screen of HIV sequence and HLA genotype data to identify HLA-mediated polymorphisms while accounting for the effect of time since infection. This is a novel way to address the question of HIV’s adaptation to HLA alleles that takes advantage of a large and well-described cohort of persons living with HIV-1 in Switzerland. This is an interesting study that provides a useful methodology for the field.

R1.1: Thank you for the positive assessment of our work.

The figures are data-heavy but sufficiently clear. My main comment is about the interpretation of the findings. I think the results highlight a limited effect of HLA-dependent selection. While I understand why the authors may not have wanted to emphasize results perceived as negative, I think it would have been useful to discuss further what the small number of significant associations vs. the large number of potential associations mean.

R1.2: Thank you for raising this concern. We have revised the manuscript to address these concerns (see specific points R4.2.1 and R4.2.2 below). We have changed the wording in the abstract and added a paragraph to our discussion section. While we now try to provide a more balanced message, we also want to emphasize that it is not surprising that only a small fraction of HLA-HIV variant combinations correspond to escape mutations, and we do not interpret this as a negative result (see response R4.2.2). However, as noted below, we now emphasize that only a fraction of HLA-mutation pairs with a signature of selection show an effect on viral load.

Reviewer #2: This study took a novel approach to enable a comparison of a very large population of individuals enrolled in the Swiss HIV cohort study to assess the host-virus interactions, by a clever utilization of the average pairwise viral diversity (APD) as a proxy for time since infection. They were able to look for associations in amino acid mutations across the HIV proteome whole viral genome sequences and (HLA) genotype and viral RNA load (VL) during untreated chronic infections. They found 98 HLA/viral-variant pairs over time, 12 of which associated with an impact on VL. 48/98 were supported by computational HLA-epitope predictions.

This is an valuable approach for studying the impact of CD8 T cells, the statistical strategies are appropriate, and as they note in the discussion, “This approach stands out by harnessing the potential of cross-sectional data and hence bridging between the within-host and epidemiological perspectives.”

While paper seems elegant in terms of bioinformatics, the authors could improve the paper by providing more biological context from the literature regarding their most interesting associations.

R2: Thank you for the positive feedback and for addressing this point. We have now added more information to the epitopes and their biological meaning as outlined below (R7.4).

Reviewer #3: This is an interesting analysis in which the authors assess potential time-dependent effects of HLA-associated HIV adaptation using cross-sectionally sampled near full length viral genomes.

The authors emphasize their development and use of a viral genetic diversity score (average pairwise diversity, "APD") as a proxy for time since infection. While this measure appears to be useful, as stated, because HIV genetic diversity does tend to increase over the course of untreated infections, I am bit puzzled by the authors' insistence that this approach and measurement is such a novel development. From the paper it seems (to this reviewer, at least) that the authors are focused on the APD itself, as the major new tool, rather than the fact that this tool is required only because the authors are attempting to use single timepoint samples from individuals without good data on time since infection in order to infer time since infection. And that once individual time since infection can be inferred, or APD used as a proxy, then longitudinal patterns of HIV evolution can be identified. I might even recommend a different title that highlights that approach, rather than the generally descriptive current title. Something along the lines of, "Using intra-host genetic diversity to approximate time since infection in studies of HIV and HLA interaction."

R3.1: Thank you for your feedback and suggestions. We would like to clarify that while our work does not introduce APD as a novel concept, its application within the context of identifying viral mutations under HLA-mediated selection is relevant and novel. Previous studies, such as Puller et al. (2017) and Carlisle et al. (2019), have validated APD as a reliable proxy for time since infection (TSI) in people living with HIV (PLWH). Given the lack of precise TSI data for most PLWH and very few longitudinal data off antiretroviral treatment in current studies, utilizing APD as a proxy allows us to infer TSI and detect time-dependent trends using robust cross-sectional data from over 1000 participants. We have integrated a new paragraph into the introduction (please refer to point R4.3), where we delve into the correlation between APD and TSI and discuss its practical implications.

Regarding the suggested title, “Using intra-host genetic diversity to approximate time since infection in studies of HIV and HLA interaction,” while informative, it may not fully encapsulate the breadth of the research included in this article: Our study extends beyond focusing on the APD as an approximate for time since infection, and present significant methodological advancements and novel findings. We now specifically highlight HLA-HIV pairs that were not previously described in the literature (to our knowledge) in Table S2 and in the discussion lines 366-373 (see point R3.2).

Including cross-sectional and longitudinal analyses in this paper does fill out the scientific story well.

Overall, with regards to findings, the results are broadly confirmatory, in that HLA escape mutations decline in the absence of the specific HLA and increase in the presence of the specific HLA, and B-5701 is, once again, shown to be the HLA with the strongest impact on HIV disease (VL) and viral evolution. Thus it is slightly a methods paper, rather than a findings paper, and this perhaps should be the emphasis.

R3.2: We are aware that this paper has a strong focus on methods. However, based on these methods it also derived new results: The approach allows for a systematic and comprehensive assessment of interactions between viral mutations and the viral genome which also results in 32 novel, previously unidentified pairs (see Table S2). Our goal is to balance both aspects and to ensure that each receives appropriate attention. To highlight our findings and the benefits of such a large-scale systematic genome-wide screen, we added a new paragraph to the discussion. We hope the additional paragraph will demonstrate that the new findings reported here are not simply the validation of previously established methods.

Line 366-373: “A comparison of our findings with those of previous studies and the Los Alamos Immunology database revealed that 32 of the 98 pairs identified through our time-dependent analysis were to our knowledge not previously described (S2 Table) and are not included in [12, 13, 33, 34]. This may be attributed to the fact that our systematic screen had a high sequence coverage across the whole HIV genome, even short genes like vif, vpr, tat, and rev. Additionally, we included HLA class II alleles in our analysis. However, it is important to note that these associations should be interpreted with caution, as they may be the result of linkage disequilibrium, as mentioned in the previous paragraph.”

**Part II – Major Issues: Key Experiments Required for Acceptance**
Please use this section to detail the key new experiments or modifications of existing experiments that should be absolutely required to validate study conclusions.

Generally, there should be no more than 3 such required experiments or major modifications for a "Major Revision" recommendation. If more than 3 experiments are necessary to validate the study conclusions, then you are encouraged to recommend "Reject".

Reviewer #1: It appears that the statistical analysis is based on the Ia dataset, ie a dataset that has a third of participants who are on ART. While I understand that this cohort can be used to look for associations between HIV sequences and HLA alleles, it is problematic to use this dataset for the time dependent analysis. Because the effect of time is measured using a proxy metric that corresponds to diversity at the third codon position, it seems important that this measure be calculated on sequences isolated from participants with uncontrolled viremia. Unless all the participants on ART are failing therapy, I think the participants on ART should be excluded and the analysis to look for associations should be based on the Ib dataset of ART naïve individuals.

R4.1: We fully agree with the reviewer that the main analysis should only be applied to treatment-naive individuals, and indeed this was also how we performed the analysis, i.e., it was not done in statistical analysis group Ia but in Ib (as the reviewer suggests). Our original wording may have been unclear in this regard, and we have now clarified this in both the methods and results sections as follows.

Line 451-452: “…, all assessments utilizing APD are limited to sequences obtained prior to ART initiation (participants in statistical analysis Ib, see Fig 1).”

Line 472-474: “APD was only added to the models in analysis Ib and not Ia, as the participants needed to be ART-naïve.”

The results did not lead me to the same conclusions as the authors. I see limited effects with no association for 335 HIV/HLA pairs and only 98 associations, including just 12 with an effect on viral loads. In contrast, the abstract mentions ‘numerous novel interactions’ and ‘frequent HLA-mediated selection’. It would be useful give more precise numbers for what numerous corresponds to as the results highlighted only a few examples. I think the ‘textbook’ scenario of escape mutation followed by a viral load effect has been clearly demonstrated in only a few cases in the literature, and I felt that the data supported that this is indeed a rare scenario and that in most cases the interactions between sequence/HLA diversity, time and viral loads are complex and difficult to identify.

R4.2.1: We understand that there may be a discrepancy between the perceived importance of our findings and the actual numerical data presented. However, we also believe that this is not unexpected or concerning. In light of the referee's concern, we have adapted the wording in the abstract to take this into account:

Line 14-17: “Our diversity-based approach allows a powerful and systematic investigation of the interaction between the virus and cellular immunity, revealing a notable subset of interactions. From an evolutionary perspective, these observations underscore the complexity of HLA-mediated selection pressures on the virus that shape viral evolution and pathogenesis.”

R4.2.2: Furthermore, we aim to provide a more nuanced assessment of the biological relevance of our results and their implications for VL in the discussion. It is important to note that absence of evidence is not evidence of absence. It is also not unexpected that mutations affecting viral fitness may have weak, and often statistically non-significant effects on VL (see Bonhoeffer et al. (2003)).

Line 313-327: “The identification of "only" 433 HLA/HIV-variant pairs out of over two million possible combinations may appear modest at first glance. However, it is important to note that it is to be expected that only a minority of positions in the viral genome interact with HLA, due to the selectivity of HLA binding, immuno-dominance, restriction by the proteasome, and T cell receptors, as well as constraints to viral evolution [6, 9]. Moreover, our analysis revealed that even fewer pairs exhibited a time-dependent relationship (98/433), with an even smaller subset demonstrating a significant effect on VL (12/98). These findings underscore the complexity of the host-virus interaction landscape, and HIV's ability to adapt and evolve in response to immune pressure. This is particularly evident in the observation that for the majority of variants identified to be under HLA-dependent selection in analysis Ib (86/98), the interaction effect between HLA and viral variants on VL was statistically insignificant, and they did not exhibit the anticipated virus-load pattern typically associated with escape mutations. This observation may be attributed to limitations in statistical power. However, it is also consistent with previous analyses indicating that moderate differences in viral fitness may often result in no or only weak variations in VL [13, 28].”

It would be useful to provide more details on the APD and specifically on how it relates to time since infection as previously described in Carlisle et al. and in the context of the current lengths of infections in the participants studied.

R4.3: We agree that we should have included more information about the APD. To address this, we have expanded the paragraph in the introduction. Please note that the duration of infection reported by participants does not provide any new information.

Line 58-67: “Based on comprehensive data from the Swiss HIV Cohort Study (SHCS)[21], we applied a previously established and validated viral genetic diversity score (average pairwise diversity, APD [22, 23]) as a reliable proxy for time since infection (TSI). Determining the TSI can be challenging as people may live with HIV for many years before being diagnosed [22, 23]. HIV diversity increases throughout the infection, and this diversity can be used to derive the TSI. Of several methods that utilize viral diversity, APD was characterized by a mean absolute error of less than one year and higher sensitivity and specificity than, for example, the fraction of ambiguous nucleotides, FAN, method [22, 23]. We used APD to systematically assess the time-dependence of HLA/HIV-variant associations. This has allowed us to gain novel insights into the evolving relationship between the human immune system and the ever-adapting HIV.”

Reviewer #2: I think they should provide background to validate that the HLA associated mutations are in actual epitopes when possible, by comparing more directly to the literature. Though the predictions they have implemented are useful, more should be done. I give some examples in Part II for an easy way to approach this starting with the Los Alamos HIV database. In the process of doing this they may gain some insights into the proposed escape mutations they observe and the biological outcomes of responses to particular epitopes. They could do this systematically for all of Table S1, but in particular, it would be helpful to draw out more cleanly and detail what is known regarding the 12 examples that were associated with VL differences would be valuable. I think this would make the paper both more readable and interesting to the immunology community.

R5: Thank you for bringing this point to our attention. We agree that this should be added to Table S1. We have done this as described below in R7.4 (see also S2 Table).

Reviewer #3: (No Response)

**Part III – Minor Issues: Editorial and Data Presentation Modifications**
Please use this section for editorial suggestions as well as relatively minor modifications of existing data that would enhance clarity.

Reviewer #1: Abstract line 12: should it be 28 of 58 instead of 28/98 as described in the results (given that 40 pairs were excluded for lack of events)

R6.1: Thank you, we adapted the sentence as follows:

Line 12-13: “Furthermore, 28/58 pairs were validated by time-to-event analyses and 48/92 by computational HLA-epitope predictions.”

Line 66- Table 1- Figure 1: the numbers do not match between the table, figure and text. It would be useful to describe what the analysis numbers (eg Ia) correspond to the first time they are described or the corresponding numbers are described.

R6.2.1: We understand that this is very confusing. We have adjusted Table 1 to improve readability (added boldface for numbers that refer to main analyses, and added more information in the table legend), and we have revised this sentence in the results:

Line 70-72: “Among all SHCS participants who met our selection criteria (Fig 1A, Table 1), 1,044 had at least one ART-naïve HIV sample sequenced and their HLA alleles genotyped (analysis Ib), and 829 had VL measurements within 180 days around the sample date (analysis II).”

Line 90-92: “Table 1: Characteristics of SHCS participants included. The number of participants used for cross-sectional (I and II) and longitudinal analysis (III) and their characteristics are shown for each analysis. Numbers in bold (subtype B) refer to the number of participants included in the main analyses (I-III).”

R6.2.2: Additionally, we added the number of participants to the results for each analysis:

Line 126-128: “Among the 1,044 SHCS participants included in statistical analysis Ib, we found 98 significant HLA/HIV-variant pairs among all 433 pairs with significant associations in Fisher's exact tests (Fig 2A, S2 Table).”

Line 163-164: “Assessing those effects among all 98 pairs with significant interaction effects between APD and HLA on the viral variant in analysis II, we …”

Line 193-194: “Using longitudinally sampled HIV sequences, we evaluated the impact of HLA alleles on the hazards of HIV variant occurrence in analysis III.”

Line 91 + discussion. The discussion describes effects in the different genes and that associations in some genes appear to be over-represented but they do not discuss why associations in Env are rare compared to the size of Env. It would be useful to describe the results for Env in more details in the paragraph starting line 91. Could the authors also provide more details on the sequence coverage of each gene, i.e. did all participants have env sequences used in the analysis?

R6.3: Thank you for pointing this out. We added a supplementary table with information on sequence coverage (see Table S1) and we adapted the manuscript in the following sections:

Line 112-113: “The protein Env had the lowest relative frequency (41/930’696) (S1 Table).”

Line 349-358: “Even though we had good sequence coverage of Env in our study, the number of HLA-HIV associations was relatively low compared to the number of possible combinations. This can be attributed to several factors. For one, Env is highly variable, with three times more possible HLA/HIV-variant combinations than for example Pol, thus it may have fewer stable and conserved epitopes that can be presented to HLA molecules. Furthermore, much of the selection pressure on Env comes from antibody escape, so the lack of detection of HLA class I-dependent effects could be due to the fact that HLA class I-dependent selection plays a weaker role compared to other HLA class II or HLA-independent mechanisms [31, 32]. This may result both in a lower number of HLA-HIV interaction pairs and a lower statistical power to detect them.”

Lines 113-125: How were the 8 associations described in Figure 2B selected?

R6.4: They were chosen to be representative of the significant pairs.

Line 155. Verify the IQR value (maybe 1.02 instead of 2.02].

R6.5: Thank you for pointing that out. We adjusted the IQR values.

Line 200-202: “The median time to mutation was shorter in the presence of HLA (median [IQR]: 1.36 [1.04, 1.65] years) compared to the participants without the HLA allele (median [IQR]: 1.44 [1.29, 1.48] years).”

Line 178-180: For the computational epitope predictions, is there a possibility to use a continuous measure (or fold change) to see if there is a difference associated with a mutation that might be smaller than what is seen with the categories.

R6.6: Thank you for your suggestion. The EL Rank score is more accurate (higher sensitivity and specificity) than a continuous measure because it corrects for the imbalance in the prediction score. Therefore, we decided to use the rank system. However, for comparison with other models, e.g. HLA-APD, we used a rank ratio (to make it more continuous). So it is not really possible to use a more continuous measure than what we have already tried.

Figure 2C: typo in ‘occurrence’ in the y axis label

R6.7: Thank you for pointing that out. We corrected it.

Reviewer #2: 1. These two papers would be worth mentioning in the introduction. While the McMichael group did a genome wide analysis with longitudinal samples and very detailed experimental validation of the variants and CTL responses that were observed early in infection, such experiments are constrained by their complexity to small number of people, and the less detailed broader view is of course also of interest. They might consider exploring the relative entropy of the mutations they observe associated with HLA and viral load, building on what Liu et al. observed.

Vertical T cell immunodominance and epitope entropy determine HIV-1 escape. Liu MK et al. J Clin Invest 2013 Jan;123(1):380-93. doi: 10.1172/JCI65330. Epub 2012 Dec 10. PMID: 23221345

The first T cell response to transmitted/founder virus contributes to the control of acute viremia in HIV-1 infection. Goonetilleke N et al., J Exp Med. 2009 Jun 8;206(6):1253-72.
doi: 10.1084/jem.20090365. Epub 2009 Jun 1.
PMID: 19487423

R7.1: Thank you, we now mentioned the two papers in the introduction:

Line 50-54: Previous longitudinal studies described HIV escape mutations occurring within three years after infection. In the presence of a “protective” HLA allele, mutations occurred more rapidly, and in its absence, they reverted quicker upon transmission [16-20]. These studies, however, focused on specific viral epitopes in small populations, therefore lacking the systematic approach of the genome-wide screens.

2) Line 118: How do they interpret negative interaction terms? (line 118)

R7.2: In the presence of a specific HLA allele, the corresponding HIV variant is less likely to occur with an increase in APD (i.e., over time). It is explained in more detail with the example of Rev-G57E both in the results (lines 154-156 and 174-177) and the discussion (lines 306-312).

3) Line 127 and 130: Regression models, HLA-B*57:01 and B*57:03 were highly associated with lower VL. This was the strongest interaction, and needs to be put in a better literature context, as do the full set of 12 HLA/HIV-variant pairs in total with a significant interaction effect on VL.

R7.3.1: We appreciate your observation regarding the absence of a literature context. In order to provide a more comprehensive understanding of the protective alleles and the 12 pairs, we have expanded the two paragraphs in the discussion as follows:

Line 291-312: “Twelve HLA/HIV-variant pairs had a significant HLA-dependent effect on VL and the magnitude of their effect on VL was strongly correlated with the magnitude of the HLA-dependent selection inferred in the diversity-based screen (ρ=0.94, p<0.001). Most (11/12) of these interaction effects resulted in increased VL, consistent with previous findings [12, 13] and their classification as HLA-escape mutations. The increase in VL can be explained by the fact that HLA alleles such as B*57:01, B*57:03, or C*12:03 lose their "protective" effect (lowering VL) when an escape mutation like Gag242N occurs [12, 13, 15]. This is especially relevant when the escape mutation has low fitness costs or reversion rates, as we saw for example with Vif33R and B*57:01 (Figure 2D, Figure 3D). At the population level, this could enhance infectivity and pathogenicity [25, 26], similar to what was described in a Japanese study with homogeneous A*24:02 distribution selecting for the escape mutation Nef135F [27].

Of the twelve pairs that significantly interacted with VL, only the two pairs Vif33R:B*57:01 and Pol432R:A*03:01 were previously described as significantly lowering VL [12, 13]. For all others, this interaction effect on VL was either not significant (Gag-264K, Pol54L, Pol68P, Pol91D, and Tat32L) [13] or never identified before (Pol9L, Rev57E, and Nef102H). Conversely, for the newly identified pair Rev57E:B*40:01, we observed an HLA-dependent decrease in VL upon mutation. Rev57E accumulated over time in the absence of B*40:01 but disappeared in its presence, and binding predictions indicated that Rev57E induced a shift from non-binding to strong binding to B*40:01. This suggests that Rev57E increases viral fitness in the absence of B*40:01 but exposes a B*40:01-dependent epitope causing a fitness cost in the presence of B*40:01. These findings offer insights into potential epitopes with high mutational barriers and/or deleterious mutations, which may warrant attention in vaccine design efforts.”

Also, they say the 12 cases are illustrated in Fig. 3D and 3E, and perhaps I am missing something but I don’t see 12 highlighted in Fig. 3D and 3E, and I’m not getting how to link between these figures and the table S1, where 12 they are noted.

A think a figure that really specifically highlights the 12 cases would be helpful.

R7.3.2: Thank you for this suggestion. The 12 are highlighted by their significance as whole symbols in Figures 3D and 3E. As this was indeed not clear enough, we specified it in the Figure 3 legend:

Line 180-190 and Figure 3 legend: “Fig 3: Viral load association with human leukocyte antigen and HIV variant interaction. A) Multivariate linear regressions of eight HLA/HIV-variant pairs of interest (same as in Fig 1B), depicted as their association and interaction effect on viral load (VL). B, C) VL distribution over four groups for pair Pol432R:A*03:01 (B) and Rev57E:B*40:01 (C). The absence of human leukocyte antigen (HLA) or HIV variant is coded as ‘0’ and the presence as ‘1’. Each participant is represented by one point (mean log_10_ VL), and violin and boxplots show distribution and group median. Coloration according to panel A. D, E) Multiple linear regression estimates of HIV amino acid (AA) variant on VL (D) or HLA allele on VL (E) and HLA/HIV-variant interaction on VL plotted for Gag242N:B*57:01 and all 98 HLA/HIV-variant pairs with significant average pairwise diversity (APD)-HLA interactions (analysis Ib). The 12 HLA/HIV-variant pairs with a significant interaction effect on viral load (*p<*0.05) are represented by whole symbols and all non-significant (n.s.) by empty symbols. Shapes indicate HIV protein. Coloration according to panel A. Upper-left section in E (yellow) indicates expected HLA escape mutations.”

4) Line 166-181 and Table S1: this is a more detail relevant to the point also mentioned in Part II:

“We used computational MHC class I binding prediction, to assess whether the identified HLA-1
allele/viral-variants pairs could be explained mechanistically”.

It would be helpful to list the specific proposed epitopes that changed HLA binding based on epitope prediction to the table. They check all 9 mers spanning the site of interest, but what do they think is actually the likely epitope(s) based on the EL predictions? Could they write them out?

As the binding predictions are not really validation, rather just support for the epitopes overlapping the variable sites, further validation with known experimentally defined epitopes from the literature whenever possible would provide a clearer proof.

So I think Table S1 would benefit from more information added, or alternatively an additional table could be added.

A quick way to begin to explore this literature would through the immunology database at Los Alamos, where they could cross-check their observed HLA/mutational associations and predictions against both known epitopes and experimentally determined escape patterns.

An easy way to at least begin to look more deeply is to compare supplemental table S1 to the epitope maps at the Los Alamos database. I think they would find examples of mapped epitopes, escape mutations, and responses associated with clinical outcomes that might enrich the context of mutation listings in their paper.

The clickable known epitope CD8 T cell maps can be found here:
<https://www.hiv.lanl.gov/content/immunology/maps/maps.html>

Here is Gag:
<https://www.hiv.lanl.gov/content/immunology/maps/ctl/Gag.html>

Their very first Table S1 entry, Gag18R:A*30:01 has an interesting characterization in previous literature:
Gag18R:A*30:01

Clicking on the epitope map takes you to this link:
<https://www.hiv.lanl.gov/mojo/immunology/search/ctl/results.html?protein_name=Gag&start=18&end=26&hla_id=467>

But a better example is a little further down the table, Gag264K~B*27:05, is embedded in a very interesting very well studied well studied B*2705 epitope. Their Gag264K is known to be associated with partial escape.

<https://www.hiv.lanl.gov/mojo/immunology/search/ctl/results.html?protein_name=Gag&start=263&end=272&hla_id=3161>

R7.4: Thank you very much for this helpful comment and the useful information and links. We have now added more published experimental data and epitope characterizations to support our findings in several places in our manuscript.

First, all epitopes used for NetMHCpan have been added to Table S2 (formerly Table S1). In addition, all pairs and their corresponding characterization in the Los Alamos database are highlighted and noted in the reference list.

Second, we refer to the table in the methods and have expanded the following sentence in the methods section:

Line 224-227: “Among the 92 pairs tested (excluding MHC class II, n=6), 66 pairs (98 epitopes) were predicted to exhibit at least "weak" binding of at least one non-mutated or mutated 9-mer epitope to the corresponding HLA allele (see S2 Table for more information on epitopes).”

Finally, we explicitly refer to Table S2 in the discussion to highlight which HLA/HIV-variant pairs represent novel findings and which have been previously described and studied experimentally (see response R3.2). We hope that these changes provide the reader with the necessary biological context and experimental epitope confirmation from the literature.

5) Line 328. “For the translation and the multiple sequence alignment of these amino-acid sequences, the HIV-1 HXB2 reference genome (GenBank accession number K03455.1) was used. alignment was done separately for each analysis and protein using MAFFT 7.520(48) an MUSCLE 3.8.31(49)."

Is HXB2 also the numbering system you used to describe mutations?

R7.5: Yes, exactly. In order to make it more clear to the reader, we have added this information to methods:

Lines 434-438: “For the translation and the multiple sequence alignment of these amino-acid sequences, the HIV-1 HXB2 reference genome (GenBank accession number K03455.1) was used. The nomenclature for HIV amino acid variants presented in this study follows the HXB2 numbering system. The alignment was done separately for each analysis and protein using MAFFT 7.520 [54] and MUSCLE 3.8.31 [55].”

6) Are the HIV sequences all submitted to Genbank? I didn't see the accession numbers, excuse me if this was noted and I missed it. A full alignment of all sequences to accompany the paper would also be helpful.

R7.6: We value your suggestion to submit our sequences to GenBank. Regrettably, due to the Swiss HIV Cohort’s regulations, we are unable to do so at present (for more details, please refer to their statement below). We acknowledge the value of a full alignment and are considering future possibilities within these constraints.

Open data statement SHCS:

The individual level datasets generated or analyzed during the current study do not fulfill the requirements for open data access:

1) The SHCS informed consent states that sharing data outside the SHCS network is only permitted for specific studies on HIV infection and its complications, and to researchers who have signed an agreement detailing the use of the data and biological samples; and
2) the data is too dense and comprehensive to preserve patient privacy in persons living with HIV.

According to the Swiss law, data cannot be shared if data subjects have not agreed or data is too sensitive to share. Investigators with a request for selected data should send a proposal to the respective SHCS address ([www.shcs.ch/contact](http://www.shcs.ch/contact)). The provision of data will be considered by the Scientific Board of the SHCS and the study team and is subject to Swiss legal and ethical regulations, and is outlined in a material and data transfer agreement.

This statement is a direct copy from <https://www.shcs.ch/294-open-data-statement-shcs> (05/2024).

7) To name their mutations they provide a number with an amino acid, but could they add the amino acid before and after a change? Is Gag18R actually Gag K18R, and Gag264K actually Gag R264K for example? If so, that kind of label would be clearer.

R7.7: Thank you for your suggestion. We agree that this K18R format may enhance the clarity of the text for readers. However, to maintain the concision of the (already dense) figures while avoiding potential confusion, we decided to retain the 18R format in the main text and figures (similar to what was done by Gabrielaite et al. (2021)) and to include the proposed K18R notation in Table S2. The table now lists the corresponding amino acids using the population consensus for each mutation discussed. This allows readers to compare our findings with those of other studies.
