# Supplementary material for: Using viral diversity to identify HIV-1 variants under HLA-dependent selection in a systematic viral genome-wide screen
Source: PLoS Pathog. 2024 Aug 8;20(8):e1012385. doi: 10.1371/journal.ppat.1012385 (PMC11335148; doi:10.1371/journal.ppat.1012385)
Supplement: Attachment — Submitted filename: letter_responses_review_2.docx [file ppat.1012385.s012.docx]

Dear Editor

We submit herewith a revised version of our manuscript. We are grateful for the reviewers' constructive comments, which we addressed in detail in our point-by-point reply below.

Throughout the point-to-point reply, we use the following color code:

R: Our responses are marked in blue.

Line x-y: Modifications to the manuscript text are marked in red.

Thank you for reconsidering our paper for publication in PLoS Pathogens.

On behalf of all authors,

Nadia Neuner-Jehle and Roger Kouyos

Each of the reviewers focused on a different issue in your paper. You should carefully consider each of these comments in a revision.

**Part I - Summary**
Please use this section to discuss strengths/weaknesses of study, novelty/significance, general execution and scholarship.

Reviewer #1: The revised version of the manuscript is improved. However, when rereading it, there are still things that are confusing regarding which participants are included in which analyses. It is unclear why they report the number of non-B sequences in Table 1 if the analyses are only done on a subtype B dataset. Could this information be in a Supplementary Table?

R1: Thank you for bringing this to our attention. We have made the necessary adjustments to Table 1 to enhance its clarity. All the data related to non-B subtypes has been transferred to the S3 Table, where it is referenced in the section on the sensitivity analyses.

Line 252-256: “Whilst inferring HLA-APD interaction effects for 320 HIV-1 non-B subtype sequences (see S3 Table), we observed a moderate correlation with the effects for subtype B (ρ=0.34, p=0.005; S5E Fig) and saw that five pairs retained significance, reflecting potential subtype differences, and limited statistical power for non-B subtypes.”

It would be helpful to provide a definition of variant in their context. Based on the figure and on the description of ‘combinations of HLA alleles and HIV amino acid variants’, I had understood that they were identifying pairs as corresponding to an HLA allele and a residue X at specific position. However, the text on page 8 line 105 could suggest an alternate meaning: ‘Of the 433 remaining significant pairs, 329 showed a positive association, wherein the viral variant was more prevalent when the HLA allele was present (OR median [IQR]: 2.86 [2.19, 3.83], as shown in S2 Fig).’ This statement suggests that there is a reference to define a variant. Is it the consensus at each position that is the reference? Is it HXB2? Are they comparing the most common variant, e.g.. a variant that corresponds to the residue A to all the non-A variants for a given site+HLA?

R2: Thank you for bringing this to our attention. Indeed, we compare the most common variant (residue A) to all the non-A variants as suggested. As this was not sufficiently explained in the manuscript, we have added the following paragraph to the methods section:

Line 491-493: “In all statistical analyses I-III, the viral variant was compared to the most prevalent variant at a given position. For instance, a variant corresponding to residue X was compared to all non-X variants at a given position.”

Regarding the statement ‘Ancestral and population structure biases likely accounted for these associations’, did the authors perform any phylogenetic correction ? (see Bhattacharya and colleagues (Science, 2007; <https://www.science.org/doi/10.1126/science.1131528>) who showed that most of the associations in Moore and colleagues (Science, 2002; <https://www.science.org/doi/10.1126/science.1069660>) were spurious because they were due to founder effects). The authors should address the potential need for phylogenetic correction.

R3: Thank you for raising this important point. We agree that population structure and founder effects are a potential concern for analyses testing the association between HLA alleles and viral mutations, and that phylogenetic correction, as shown by Bhattacharya et al, is a valuable method to reduce such effects. However, we chose not to include phylogenetic correction because our primary focus is on examining time-dependent signals, i.e., whether the frequency of mutations increases over the course of infection. The effects examined by the interaction term between HLA and APD and in the survival analyses are not subject to the type of confounding described above (as they do not primarily depend on the association of mutations with HLA alleles, but also consider the dynamics of a mutation within individual PWH). Since in our workflow the association between the mutation and the HLA allele is primarily used as a pre-filtering step, these lineage/founder effects could potentially explain (along with statistical power) why we lose a substantial fraction of the associations between the first two analysis steps (analysis Ia and Ib).

In addition, we included in our analysis only participants with subtype B, thus reducing the likelihood of subtype-driven associations, as previously mentioned by Bhattacharya et al. In the supplementary material, we have now also added a sensitivity analysis including all subtypes, adjusted for the first 10 viral PCs, thus accounting for the viral population structure. Interestingly, this analysis recovered almost all HLA*APD associations observed in the subtype B analysis (which was not adjusted for viral PCs), underlining the robustness of these effects against correcting for population structure in different ways (restriction to most common subtype vs. adjustment by PCs). In conclusion, it is possible that our analysis Ib contains spurious associations, but it is unlikely that this confounding remains in our main analyses (Ib, II, and III). We have therefore added the following clarifications to the result and discussion sections of our manuscript:

Lines 249-263: “As statistical analysis Ib was restricted to HIV-1 subtype B and included proviral sequences, we tested in a sensitivity analysis the impact of subtypes and sequence origin on the HLA-APD interaction for the 98 identified pairs. When excluding the 61 proviral DNA sequences, we observed almost identical results (*ρ_spearman_*=0.96, *p<*0.001; S5D Fig). Whilst inferring HLA-APD interaction effects for 320 HIV-1 non-B subtype sequences (see S3 Table), we observed a moderate correlation with the effects for subtype B (*ρ*=0.34, *p=*0.005; S5E Fig) and saw that five pairs retained significance, reflecting potential subtype differences, and limited statistical power for non-B subtypes. When the sequences of all subtypes (B and non-B) were pooled, a strong correlation was observed with the effects for subtype B (ρ=0.88, p<0.001; S5F Fig). In the pooled analysis, the majority of associations (80 of 98) from the subtype B-only analysis (Ib) were recovered. All 80 pairs exhibited significant HLA-APD interaction effects even after adjusting for the first ten viral PCs, thereby accounting for viral population structure. Lastly, HLA alleles associated with the same viral variant were examined in multivariable logistic regressions to test for the effect of linkage disequilibrium among HLA alleles. In these analyses, none of the class II HLA alleles retained their significant interaction with APD (S6 Fig).”

Lines 316-331: “The identification of "only" 433 HLA/HIV-variant pairs out of over two million possible combinations may appear modest at first glance. However, it is important to note that it is to be expected that only a minority of positions in the viral genome interact with HLA, due to the selectivity of HLA binding, immuno-dominance, restriction by the proteasome, and T cell receptors, as well as constraints to viral evolution [6, 9]. Moreover, our analysis revealed that even fewer pairs exhibited a time-dependent relationship (98/433), with an even smaller subset demonstrating a significant effect on VL (12/98). It is possible that some of the 433 pairs may be due to spurious associations resulting from population structure and founding effects [28]. However, such confounding by population structure cannot explain the HLA-APD interactions observed in 98 pairs (analysis Ib) and the effect of HLA in within-patient survival analyses (analysis III), as these reflect HLA-dependent impacts of time since infection on mutation frequency. This robustness is underlined by the fact that most of these pairs are robust to adjustment for viral population structure (S5F Fig), and hence it represents an additional advantage of the viral diversity-based method established here. In fact, this confounding may be one potential additional reason behind the discrepancy between the number of pairs (n=335) lost from analysis Ia to Ib.”

**Part II – Major Issues: Key Experiments Required for Acceptance**
Please use this section to detail the key new experiments or modifications of existing experiments that should be absolutely required to validate study conclusions.

Generally, there should be no more than 3 such required experiments or major modifications for a "Major Revision" recommendation. If more than 3 experiments are necessary to validate the study conclusions, then you are encouraged to recommend "Reject".

Reviewer #1: (No Response)

**Part III – Minor Issues: Editorial and Data Presentation Modifications**
Please use this section for editorial suggestions as well as relatively minor modifications of existing data that would enhance clarity.

Reviewer #1: (No Response)
